# IL-17A as a Potential Therapeutic Target for Patients on Peritoneal Dialysis

**DOI:** 10.3390/biom10101361

**Published:** 2020-09-24

**Authors:** Vanessa Marchant, Antonio Tejera-Muñoz, Laura Marquez-Expósito, Sandra Rayego-Mateos, Raul R. Rodrigues-Diez, Lucia Tejedor, Laura Santos-Sanchez, Jesús Egido, Alberto Ortiz, Jose M. Valdivielso, Donald J. Fraser, Manuel López-Cabrera, Rafael Selgas, Marta Ruiz-Ortega

**Affiliations:** 1 Cellular and Molecular Biology in Renal and Vascular Pathology Laboratory, Fundación Instituto de Investigación Sanitaria-Fundación Jiménez Díaz-Universidad Autónoma Madrid, 28040 Madrid, Spain; vanessa.marchant@uam.es (V.M.); antoniotemu@gmail.com (A.T.-M.); laura.marqueze@quironsalud.es (L.M.-E.); rrodriguez@fjd.es (R.R.R.-D.); luciatejedors@hotmail.com (L.T.); l.santoscorreyero@hotmail.com (L.S.-S.); 2 Red de Investigación Renal (REDINREN), Instituto de Salud Carlos III, 28029 Madrid, Spain; srayego@fjd.es (S.R.-M.); aortiz@fjd.es (A.O.); valdivielso@medicina.udl.cat (J.M.V.); mlcabrera@cbm.csic.es (M.L.-C.); rafael.selgas@salud.madrid.org (R.S.); 3 Vascular and Renal Translational Research Group, Institut de Recerca Biomèdica de Lleida (IRBLleida), 25198 Lleida, Spain; 4 Renal, Vascular and Diabetes Research Laboratory, Fundación Instituto de Investigación Sanitaria-Fundación Jiménez Díaz-Universidad Autónoma Madrid, 28040 Madrid, Spain; JEgido@quironsalud.es; 5 Spanish Biomedical Research Centre in Diabetes and Associated Metabolic Disorders (CIBERDEM), 28029 Madrid, Spain; 6 Wales Kidney Research Unit, Division of Infection and Immunity, School of Medicine, College of Biomedical and Life Sciences, Cardiff University, Cardiff CF14 4XN, UK; FraserDJ@cardiff.ac.uk; 7 Molecular Biology Centre Severo Ochoa (CBM-SO), Spanish Council for Scientific Research (CSIC), Universidad Autónoma de Madrid, Campus de Cantoblanco, 28049 Madrid, Spain; 8 Research Institute of La Paz (IdiPAZ), University Hospital La Paz, 28046 Madrid, Spain; 9 ISRIN (Instituto Reina Sofia de Investigación nefrológica), 28003 Madrid, Spain

**Keywords:** Interleukin-17A, peritoneal dialysis, chronic kidney disease, inflammation, membrane failure, mesothelial, renal, pathology damage

## Abstract

Chronic kidney disease (CKD) is a health problem reaching epidemic proportions. There is no cure for CKD, and patients may progress to end-stage renal disease (ESRD). Peritoneal dialysis (PD) is a current replacement therapy option for ESRD patients until renal transplantation can be achieved. One important problem in long-term PD patients is peritoneal membrane failure. The mechanisms involved in peritoneal damage include activation of the inflammatory and immune responses, associated with submesothelial immune infiltrates, angiogenesis, loss of the mesothelial layer due to cell death and mesothelial to mesenchymal transition, and collagen accumulation in the submesothelial compact zone. These processes lead to fibrosis and loss of peritoneal membrane function. Peritoneal inflammation and membrane failure are strongly associated with additional problems in PD patients, mainly with a very high risk of cardiovascular disease. Among the inflammatory mediators involved in peritoneal damage, cytokine IL-17A has recently been proposed as a potential therapeutic target for chronic inflammatory diseases, including CKD. Although IL-17A is the hallmark cytokine of Th17 immune cells, many other cells can also produce or secrete IL-17A. In the peritoneum of PD patients, IL-17A-secreting cells comprise Th17 cells, γδ T cells, mast cells, and neutrophils. Experimental studies demonstrated that IL-17A blockade ameliorated peritoneal damage caused by exposure to PD fluids. This article provides a comprehensive review of recent advances on the role of IL-17A in peritoneal membrane injury during PD and other PD-associated complications.

## 1. Introduction

Chronic kidney disease (CKD) is a devastating disease that affects 5–7% of the worldwide population. Current treatments have limited effectiveness and only delay disease progression, underscoring the need to develop novel therapeutic approaches to either stop or reverse progression. Regardless of the underlying etiology, most CKD patients slowly progress to the permanent loss of kidney function characterized by progressive and irreversible nephron loss and reduced renal regenerative capacity, which is modulated by inflammation, leading to end-stage renal disease (ESRD) [1]. For this reason, until renal transplantation can be achieved, these ESRD patients need replacement therapies, such as peritoneal dialysis (PD) or hemodialysis.

**Figure 1:**
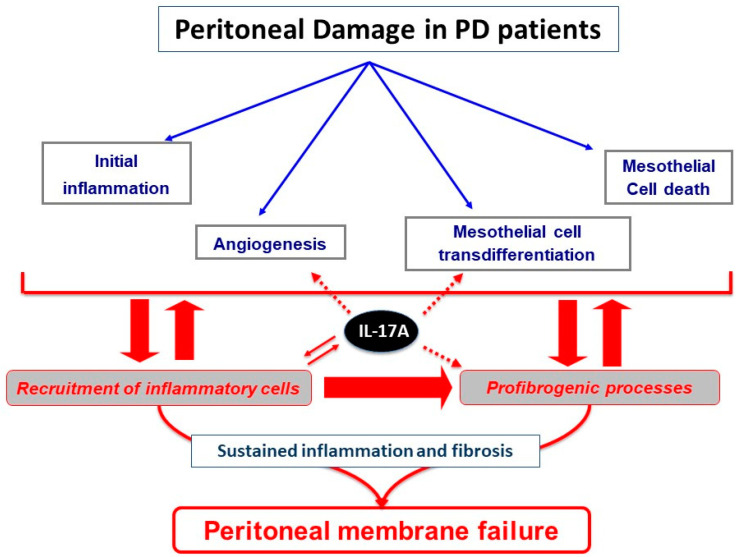
Processes involved in peritoneal damage by peritoneal dialysis: the exposure of peritoneal membrane to peritoneal dialysis (PD) treatment induces cellular and molecular responses, including inflammation, cell death, phenotype changes, angiogenesis, and submesothelial collagen accumulation, leading to membrane failure. The local production of interleukin (IL)-17A in the damaged peritoneum by immune infiltrating cells could contribute to amplification of the inflammatory response recruiting additional inflammatory cells in the peritoneal cavity. Moreover, other potential processes could be induced by IL-17A in the peritoneum, including angiogenesis, cell differentiation, and fibrosis (represented by segmented arrows).

Regarding PD, chronic exposure to conventional peritoneal dialysis fluids (PDF) has been related to peritoneal dysfunction associated with loss of the mesothelial cell monolayer, submesothelial fibrosis, vasculopathy, and angiogenesis [2,3]. Conventional PDF contain high glucose (ranging from 1.5% to 4.25%) and high lactate concentrations as an osmotic gradient enhancer and a buffering agent, respectively [4]. Importantly, glucose chemical instability during heat sterilization generates toxic glucose degradation products (GDP), including methylglyoxal; glyoxal; 3-deoxyglucosone; and above all, 3,4-dideoxyglucosone-3-ene [5,6,7]. Newer PDF, including biocompatible glucose-based solutions, icodextrin, and taurin solutions, have been developed to reduce the deleterious effects of PDF exposure on the peritoneal membrane (PM) [2,8]. The repeated exposure of the peritoneum to PDF and particularly to GDP evokes several PM cellular and molecular responses in the PM. These include increased peritoneal production of proinflammatory cytokines and advanced glycation end-products (AGEs) that induce cell death, proangiogenic factors such as vascular endothelial growth factor (VEGF) and transforming growth factor-β1 (TGF-β1), and other profibrotic factors [8,9]. This harmful environment leads to an inflammatory response, cell death, phenotype changes, angiogenesis, and submesothelial collagen accumulation (Figure 1). These phenomena lead to fibrosis and loss of PM function [2,3]. Episodes of infectious peritonitis, a common complication of PD, further contribute to these changes [10]. All these processes contribute to the loss of PM function and technique failure often found in long-term PD patients [11].

Multiple lines of evidence point to the importance of immune cells in the pathogenesis of chronic inflammatory disorders, including renal diseases and PD-related pathologies. In the peritoneal dialysate effluent (PDE) of ESRD patients exposed to different conventional PDF, elevated proinflammatory cytokine levels, together with interleukin (IL)-17A, IL-6, IL-1β, Tumor necrosis factor-α (TNF-α), and TNF-like weak inducer of apoptosis (TWEAK), have been found [3,12,13,14,15,16,17], clearly showing the involvement of inflammation in peritoneal damage induced by PDF exposure. Among these inflammatory mediators, cytokine IL-17A has recently been proposed as a potential therapeutic target for chronic inflammatory diseases [18,19].

In this review, we discuss the current data about IL-17A in ESRD patients on PD and in related preclinical studies, addressing the cellular responses and molecular mechanisms triggered by IL-17A and its potential contribution to peritoneal membrane pathophysiology (Figure 1).

## 2. IL-17A: A Key Proinflammatory Cytokine and Therapeutic Target in ESRD Patients on PD

IL-17A (usually referred only as IL-17) is the founding member of the IL-17 family of cytokines, which also includes IL-17B, IL-17C, IL-17D, IL-17E (also called IL-25), and IL-17F [20]. IL-17A is a pleiotropic cytokine that exerts mainly proinflammatory effects acting in concert with other pro-inflammatory mediators (notably, IL-1-β and TNF), although their molecular mechanisms can differ depending on cell type and pathological conditions [21,22,23,24,25]. The binding of IL-17A to its receptors IL-17RA/RC, expressed in many circulating and tissue cells, can activate several intracellular signals [26,27]. The most relevant downstream molecular pathways include mitogen-activated protein kinases (MAPKs) and the transcription factors nuclear factor-κB (NF-κB), activator protein 1 (AP-1), and CCAAT-enhancer-binding proteins (C/EBPs), leading to transcriptional activation of antimicrobial peptides, cytokines, and chemokines [28,29,30,31,32]. Firstly, we will discuss the origin of IL-17A in the peritoneum exposed to PDF and then the peritoneal responses to local production of IL-17A (Figure 1).

### 2.1. IL-17A Producing Cells: Th17 Immune Cells and Other Cells Involved in PD-Induced Damage

IL-17A is the hallmark cytokine of T helper (Th) 17 immune cells, but many other cell types produce IL-17A and their relative relevance depends on the pathological condition. IL-17A-producing cells include γδ T cells, mast cells, CD4^+^αβ T cells, invariant natural killer T (iNKT) cells, natural killer (NK) cells, CD8^+^ cells, innate lymphoid cells, and neutrophils, among others [29,33,34]. Here, we focus on the effects of these IL-17A-producing cells present in peritoneal damage (Figure 1). In peritoneal biopsies from ESRD patients, IL-17A immunostaining is found in submesothelial areas associated with the presence of inflammatory cell infiltration. These cells have been identified as Th17 lymphocytes (CD4^+^/IL-17A^+^), γδ T lymphocytes, neutrophils, and mast cells [35].

#### 2.1.1. Th17 Cells

Naïve CD4^+^ T lymphocytes can differentiate into different Th effector subpopulations, containing the effector subtypes Th1, Th2, Th9, Th17, and Th22; follicular Th; and the regulatory T lymphocytes (Treg cells) [36]. Each cell subtype is characterized by specific cytokine profiles and participates in different physiological and pathological responses, but there is a great plasticity among them not fully understood yet [37,38,39,40]. The specific Th cell differentiation is driven by the exact combination of cytokines and activation of specific transcription factors [29,41,42,43]. In particular, human Th17 cells differentiate from naïve T cells under the influence of TGF-β1 and proinflammatory cytokines (IL-1β, IL-6, and/or IL-21), mediated by the activation of the transcription factors signal transducer and activator of transcription 3 (STAT3) and retinoid related orphan receptor γt (RORγt) and their related genes including IL-23R [33,44,45,46,47]. Th17 cells secrete mainly cytokines of the IL-17 family, especially IL-17A, and other Th17-specific cytokines, including IL-22, IL-26, and chemokine C-C motif ligand (CCL)-20 [29,43,44,48,49]. Th17 cells have a critical role of in the clearance of extracellular pathogens, but they also participate in the pathogenesis of several autoimmune and inflammatory diseases, including psoriasis, rheumatoid arthritis, and multiple sclerosis [50], and other chronic diseases, like CKD and cardiovascular pathologies, such as hypertension [51,52]. In the context of the peritoneal damage, animal models of exposure to dialysis fluids showed a loss of Th17/Treg cell balance (Th17 predominance) leading to peritoneal damage during PD [53]. Increased levels of Th17 cells were also observed in the peritoneal cavity or blood in other pathologies, such as arthritis [54] and colitis [55].

#### 2.1.2. γδ T Cells

Human IL-17A-producing γδ T cells are generated in the periphery and can be recruited to and can accumulate in inflamed tissues, contributing to persistent inflammation [56]. IL-17A production by γδ T cells is involved in antifungal immunity and in the onset of autoimmune disease [57,58]. Interestingly, studies in murine models of hypertension identified γδ T lymphocytes as the main source of IL-17A in hypertrophic hearts [59], kidneys, and aorta [60]. Moreover, IL-17A^+^ γδ T lymphocytes were observed in the kidney of patients with hypertensive nephroangiosclerosis [61]. Currently, there are studies that identified the presence of γδ T lymphocytes in the peritoneal cavity and its relationship with Th17 response. In a study in ovarian cancer, the authors demonstrated that γδ T cells are accumulated in the peritoneal cavity in response to tumors and that these cells revealed preferential production of IL-17A [62]. In other pathologies such as Mycobacterium bovis Bacillus Calmette–Guérin infection, γδ T cells were identified as the main source of IL-17A in the peritoneal cavity during the early stages of infection [63]. Similar results were observed in response to *Escherichia coli* infection in which γδ T cells in the peritoneal cavity induced IL-17 production to mobilize neutrophils [64].

#### 2.1.3. Neutrophils

In peritoneal biopsies of PD patients, a double positive staining for IL-17A and neutrophil markers (such as myeloperoxidase) was found, suggesting that neutrophils may produce IL-17A [35]. In septic peritonitis induced by *E. coli* infection, one study showed that higher numbers of polymorphonuclear neutrophils accumulated in the peritoneal cavity of mice with a septic peritonitis episode and increased their IL-17 expression during infection [65]. However, recent data suggest that cultured human neutrophils do not express IL-17A, but that it may instead be released from neutrophil extracellular traps (NETs) [33,66]. NETs are networks of extracellular fibers composed of cell-free DNA, histones, and granular proteins, which are a central part of neutrophil host defense and inflammatory function [67]. Interestingly, there is a chemokine-dependent reciprocal crosstalk between neutrophils and Th17 cells, mainly mediated by chemokines CCL-2 and CCL-20, the ligands for chemokine C-C motif receptor (CCR)2 and CCR6 [68], suggesting an amplification of the inflammatory response. In this regard, the IL-17/C-X-C chemokine receptor (CXCR)2 pathway recruits neutrophils in breast cancer [28].

#### 2.1.4. Mast Cells

Mast cells are immune cells originating in bone marrow that mature as tissue-resident cells in mucosal and epithelial tissues, including the peritoneum [69]. IL-17A-positive mast cells may play a crucial role in several inflammatory and immune-mediated diseases and cancer [70,71,72]. However, a recent study demonstrated that primary human tissue mast cells do not produce IL-17A but capture, store, and release bioactive exogenous IL-17A [73]. As neutrophils, mast cells can release IL-17A through mast cells extracellular trap (MCET) formation [66]. Mast cells have been related to several PD-related processes, such as inflammation and fibrosis, angiogenesis, immunity against bacteria (peritonitis and sepsis), and omental tissue remodeling and cell recruitment [74,75,76,77]. Nevertheless, there is controversy about the role of mast cells (deleterious or beneficial) in these processes. Some studies suggest that mast cell impact on fibrosis and inflammation depends on the timing, strength, or type (acute or chronic) of injurious stimulus [69,78]. In rats, chronic exposure to PDF resulted in an increased number of mast cells in the omentum [79]. An upregulation of mast cells was found in patients with chronic inflammatory peritoneal diseases, including peritonitis during PD, chronic appendicitis, herniotomy, and fibrosis [80]. However, another study on peritoneal biopsies of PD patients showed a reduced number of mast cells, with no correlation with time on PD, fibrosis, number of vessels, or previous episodes of peritonitis [74]. This apparently contradictory data could be explained by patient characteristics, the particular clinical situation at the time of tissue procurement, or the PDF used, among other potential explanations also discussed by the authors [74]. Interestingly, in rats with chronic renal failure induced by 5/6 nephrectomy, the number of peritoneal mast cells was significantly increased with increased peritoneal fibrosis [81].

#### 2.1.5. MAIT Cells

Recently, a new IL-17A-producing cell type was described: mucosal associated invariant T (MAIT) cells [82]. MAIT cells, composing 10% of circulating CD4^−^ T cells in adult individuals, express one of the semi-invariant T-cell antigen receptors (TCR, vα7.2-Jα33) that relies on the identification of microbial vitamin B metabolites link to the major histocompatibility complex (MHC) class I-like molecule MR1 on antigen-presenting cells. Also, MAIT cells are characterized by high expression of the ATP-binding cassette subfamily B member 1 and antimicrobial specificity [82,83]. Several subtypes of MAIT cells have been described, but all of them are CD161^high^ IL-17-secreting CD8^+^ T cell subtypes, concluding that these cells are able to produce IL-17 [82,84]. These cells are present in peritoneal cavity during spontaneous bacterial peritonitis and contribute to peritoneal inflammation [85].

### 2.2. Role of IL-17A-Expressing Cells in CKD Patients

Independent of the cause, CKD is characterized by sustained inflammation and activation of immune cells that contribute to disease progression [1]. A pioneer study showed that the in vivo delivery of Th17 cells into mice resulted in rapid development of albuminuria, glomerular neutrophil infiltration, and increased renal CXCL1 mRNA levels [86], suggesting a key role of IL-17A-producing cells in the onset of renal damage, through inflammation modulation. Early experimental studies demonstrated activation of the Th17 immune response in immune-mediated glomerulonephritis, including anti-glomerular basement membrane disease, anti-neutrophil cytoplasmic antibody (ANCA)-associated vasculitis, and lupus nephritis [87,88]. Accordingly, patients with ANCA-associated vasculitis presented elevated serum levels of IL-17A and other Th17-related cytokines, such as IL-23 [89], whereas in ANCA-associated crescentic glomerulonephritis patients, only serum IL-17C but not IL-17A, F, or E levels were increased [90]. In addition, high serum and urine IL-17A levels were found in patients with systemic lupus erythematosus [91,92] and in other immune glomerulonephritis [93,94]. More recently, preclinical and clinical studies have shown the involvement of IL-17A in nonimmune nephropathies, including hypertensive [61,95] and diabetic nephropathy [96,97]. These experimental studies suggest a local production of IL-17A in the kidney. This cytokine can act as the IL-17RA/RC expressed on different renal cells, such as mesangial, endothelial, and vascular smooth muscle cells and fibroblasts, to induce several responses, including upregulation of proinflammatory and profibrotic factors, and phenotype changes, as described in in vitro studies, and therefore could contribute to the progression of renal damage [98]. In this sense, in human renal biopsies of hypertensive nephropathy patients, positive IL-17A cells, mainly Th17 and γδ T cells, were found [61], suggesting the involvement of IL-17A in the progression of human kidney diseases. Interestingly, circulating IL-17F has been recently proposed as a systemic inflammatory protein associated with a 10-year risk of ESRD in diabetic patients, being included in a robust kidney risk inflammatory signature (KRIS proteins) [99]. However, there is controversy about the presence of elevated circulating and urinary IL-17A levels in diabetic patients with or without renal damage [98].

PD patients present subclinical systemic inflammation, characterized by elevated levels of C reactive protein (CRP); increased proinflammatory cytokines, including the Th17-related cytokine IL-6 [100]; activated Th1/Th2 responses [101]; and deregulation in the Th17/Treg ratio [35,102]. An elevated number of circulating Th17 cells were found in ESRD and kidney-transplanted patients [103]. More recently, proteomic analyses showed that serum IL-17A levels are significantly higher in CKD patients than in healthy controls [104]. Consistent with these findings, CKD stage positively correlated with the Th17/Treg ratio and serum IL-17A levels whereas it negatively correlated with serum IL-10 levels. These correlations were most marked in patients who had the poorest response to hemodialysis and PD treatments [102].

Regarding preclinical studies, several approaches using IL-17A-deficient mice or neutralizing anti-IL-17A antibodies demonstrated protective effects in experimental renal diseases, including unilateral ureteral obstruction [105], connective tissue growth factor (CTGF)-induced renal damage [106], angiotensin II-induced hypertensive nephropathy [61], and diabetic nephropathy [96,98]. However, genetic IL-17A deficiency did not attenuate CKD following subtotal nephrectomy in mice [107]. These findings suggest that IL-17A blockade should be further investigated prior to use it as a therapeutic option in renal diseases.

### 2.3. Role of IL-17A-Expressing Cells in the Peritoneum Exposed to PDF

In recent years, evidence has emerged indicating that IL-17A-mediated inflammation could contribute to peritoneal damage in experimental models of PDF exposure [35,108,109] as well as in ESRD PD patients with long-term exposure to PDF [35]. Additionally, IL-17A is increased during peritonitis episodes [35,110], as discussed later.

#### 2.3.1. PDF-Induced Peritoneal Activation of Th17/IL-17A Axis in Preclinical Models

An early study showed that, in a murine model of chronic exposure to conventional PDF leading to peritoneal fibrosis [111], the immune response was activated; was characterized by peritoneal infiltration of Th17 and γδ T cells but no Th1 and Th2 cells; increased peritoneal expression of Th17-related cytokines, such as IL-17A and IL-6; and activated Th17-related transcription factors, including RORγt and STAT3 [35]. Importantly, peritoneal IL-17A protein levels correlated with peritoneal membrane thickness [35]. Moreover, mice deficient in CD69, a leukocyte membrane glycoprotein that modulates Th17 cell differentiation via the Janus kinase (JAK) 3/signal transducer and activator of transcription (STAT) 5 signaling pathway, exposed to PDF [112] showed hyperactivation of the Th17 response and increased IL-17A production in PDE, whereas interferon-γ (IFN-γ) and IL-4 levels were not altered. Moreover, an anti-CD69 antibody mimicked the effects caused by CD69 deficiency in mice exposed to PDF. The activated Th17 response was associated with exacerbated inflammatory and fibroproliferative responses to PDF exposure [108]. In both models of PDF exposure, the contribution of IL-17A to the peritoneal damage was demonstrated by using intraperitoneal neutralizing anti-IL-17A antibodies that decreased peritoneal fibrosis [35,108]. Furthermore, bone marrow cell transplantation combining double mutant mice deficient in Rag2 and γc combined with CD69 knockout mice demonstrated that CD69 expression in the lymphocytic rather than in the bone marrow myeloid compartment was responsible for controlling Th17 cells [108]. In the same way, in uremic mice due to subtotal nephrectomy, daily exposure to conventional PDF for 8 weeks resulted in increased CD4^+^/IL-17^+^ cells in peritoneal cavity [109]. These experimental studies support the hypothesis of a deleterious effect of IL-17A in the peritoneum by the modulation of the inflammatory and fibrotic response (Figure 1). Interestingly, this study compared conventional and low-GDP bicarbonate/lactate-based PDF, showing a large difference in inflammatory response. In uremic mice, a biocompatible low-GDP PDF increased peritoneal recruitment of M1 macrophages, with higher levels of macrophage-related proinflammatory cytokines and lower number of CD4^+^/IL-17^+^ cells, and was associated with better preservation of PM integrity [109]. Although additional preclinical studies should confirm these observations, these findings suggest that conventional PDF activated the peritoneal Th17 immune response, whereas biocompatible PDF did not.

#### 2.3.2. Local Production of IL-17A in Long-Term PD Patients

Several reports have described elevated peritoneal IL-6 levels in PD patients, and therefore, this cytokine could act as a polarizing mediator of Th-17 cell differentiation [113,114,115]. An early study in 41 PD patients showed that IL-17A levels in PDE were increased and significantly higher in long-term PD patients after 3 years of dialysis [35]. In peritoneal biopsies from these PD patients, submesothelial IL-17A immunostaining was found mainly in inflammatory and fibrotic areas and correlated with peritoneal fibrosis. By double confocal microscopy, IL-17A-expressing cells were identified as Th17 cells, γδ lymphocytes, mast cells, and neutrophils [35]. Thus, the IL-17A response seems to be implicated in peritoneal damage in PD and IL-17A levels in PDE could be used as a noninvasive biomarker of PM damage in PD patients, but larger clinical studies should be performed to validate this hypothesis.

In PD patients, biocompatible PDF preserved PM integrity [116], displaying lower levels of proinflammatory cytokines in PDEs [2,8]. As commented above, preclinical studies suggested that novel biocompatible PDF did not activate Th17 immune responses [109]. However, IL-17A levels in PDE from PD patients with novel biocompatible PDF should be evaluated.

## 3. Peritoneal Impact of IL-17A

**Figure 2:**
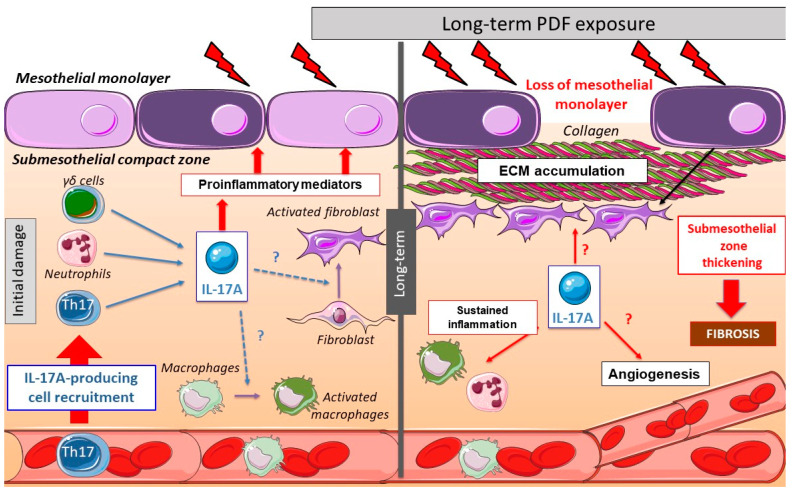
Peritoneal changes due to long-term peritoneal dialysis fluids (PDF) exposure: initially, chronic PDF exposure causes the recruitment of inflammatory cells into the submesothelial zone. Among the infiltrating immune cells, there are several IL-17A-producing cells, such as Th17 cells, γδ T cells, neutrophils, and others. The local production of IL-17A triggers the release of additional pro-inflammatory mediators by infiltrating cells and resident peritoneal cells, including cytokines and chemokines, therefore contributing to amplification of the inflammatory response. In long-term PDF exposure, the loss of mesothelial monolayer and submesothelial thickness is associated with elevated peritoneal IL-17A levels. This cytokine could also potentially promote fibrosis and angiogenesis in the peritoneum.

Few studies have explored the effect of IL-17A in vivo in the peritoneum. In mice, a single intraperitoneal administration of IL-17A resulted in a rapid increase of local levels of Granulocyte-colony stimulating factor (G-CSF) and selective neutrophil accumulation [117]. In another study, intraperitoneal IL-17A induced submesothelial inflammation, together with the presence of monocytes, CD3^+^ and CD4^+^ T lymphocytes, and neutrophils, observed at 10 days. Moreover, weekly IL-17A intraperitoneal injections for 35 days induced peritoneal fibrosis characterized by PM thickness associated with fibronectin deposition and expression of myofibroblast markers, such as fibroblast-specific protein 1 (FSP-1) and α-smooth muscle actin (α-SMA) [35]. These data suggest a direct deleterious effect of long exposure to IL-17A in the peritoneum, contributing to the peritoneal damage induced by PD (Figure 2).

### 3.1. IL-17A as a Mediator of Peritoneal Fibrosis through Activation of Inflammatory Pathways

Local tissue damage triggers an inflammatory response characterized by chemokine secretion and immune cell recruitment. Mediators secreted from immune cells eventually drive tissue regeneration and a transient local profibrotic response. However, failure of the reparative process may lead to persistent inflammation, excessive extracellular matrix (ECM) deposition, and fibrosis [27]. There is in vivo evidence supporting a profibrogenic role of IL-17A in pathological conditions associated with inflammation [118]. Thus, in cultured dermal vascular smooth muscle cells and fibroblasts from systemic sclerosis patients, IL-17A stimulated proinflammatory responses, ECM protein secretion, proliferation, and migration [119,120], supporting the profibrotic role of IL-17A.

Accordingly, the repeated exposure of the peritoneum to PDF elicits several cellular and molecular responses in the PM, including activation of an inflammatory response, production of cytokines and chemokines, and recruitment of inflammatory cells (Figure 2). As commented above, preclinical data suggest that long-term exposure to PDF induced the presence of submesothelial IL-17A-producing cells. Moreover, IL-17A could activate peritoneal cells to upregulate some proinflammatory cytokines, like IL-6 or MCP-1, which contribute to persistent inflammation. This inflammatory response could also trigger the production of profibrotic factors, indirectly contributing to fibrosis (Figure 2).

### 3.2. IL-17A in Mesothelial Cells

IL-17A binding to its receptor in mesothelial cells can induce proinflammatory responses. In cultured human mesothelial cells, IL-17A activated the canonical NF-κB pathway and downstream cytokines, including G-CSF [117] and the C-X-C chemokine GROalpha (also known as CXCL1) [121]. This proinflammatory response was increased in the presence of TNF-α [117]. In high-glucose conditions, as occurs in response to conventional PDF exposure, mesothelial cells increased the production of proinflammatory and profibrotic factors. Interestingly, high glucose activated the TLR4/MyD88/NF-κB signaling pathway to induce inflammatory mediators, as MCP-1, in mesothelial cells [122]. However, the role of TLR4 in IL-17A responses has not been evaluated.

**Figure 3:**
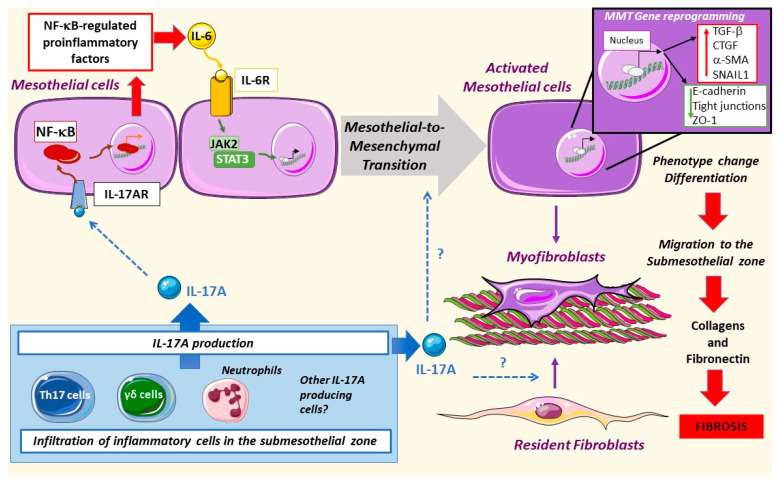
Mesothelial-to-mesenchymal transition in peritoneal damage by PDF: IL-17A produced by different cells can activate the nuclear factor-κB (NF-κB) pathway in mesothelial cells, driving the expression of regulated factors, such as IL-6. This cytokine can activate the janus kinase/signal transducers and activators of transcription (JAK/STAT) pathway leading to mesothelial-to-mesenchymal transition (MMT). Moreover, mesothelial cells change their pool gene expression as well as phenotype, increasing the motility of these cells and the deposition of collagens and fibronectin, thus promoting fibrosis. IL-17A might also activate resident fibroblasts as well as trigger MMT directly, but these processes have not been yet explored.

The exposure of peritoneal mesothelial cells to PDF results in Mesothelial-to-Mesenchymal Transition (MMT), a process characterized by phenotype alterations that induce a transition from an epithelial to a mesenchymal migrative phenotype [123,124,125,126,127]. The mesothelial cells begin to lose basolateral polarization and the expression of epithelial markers like cytokeratins, E-cadherins, and cell-junction proteins and acquire mesenchymal markers such as α-SMA, vimentin, VEGFA, Snail, collagens, and fibronectin [123,128]. Different mediators are involved in PDF-induced MMT, including AGEs [129], endotelin-1, growth factors, such as TGF-β1, VEGF, Gremlin-1 (GREM1), and CTGF/CCN2 as well as proinflammatory cytokines like IL-6 [123,124,130,131,132,133]. Although to date there is no definitive reported data, it is tempting to speculate that IL-17A may be a direct triggering stimulus of the MMT process (Figure 3).

Different signaling systems are involved in MMT. IL-17A promotes the peritoneal expression of IL-6 [35], which in a paracrine or autocrine manner binds and activates its receptor in mesothelial cells, engaging the JAK2/STAT3 pathway [114] and triggering MMT. On the other hand, TGF-β1 activation of the Smad pathway promotes the expression of profibrogenic proteins and myofibroblast markers [130,134]. AGEs activate the RhoA/Rho kinase pathway, recruiting AP-1-mediated transcription of α-SMA. This pathway is active in human peritoneal mesothelial cells as demonstrated by using inhibitors of the RhoA/Rho kinase (Y27632) and curcumin, a compound that has been shown to inhibit AP-1 [129]. The production of reactive oxygen species (ROS) is also involved in MMT [135,136]. IL-17A activates these signaling systems, including RhoA/Rho kinase, ROS production, and the MAPK cascade in different cell types, such as vascular smooth muscle cells [30,31,137,138,139], but data on mesothelial cells are lacking.

A majority of MMT responses converge in Snail expression, which is the principal driver of mesothelial cell junction disruption and loss of basopolarity, leading to a mesenchymal phenotype [128,140]. Among others, Snail expression is regulated by NF-κB, illustrating the key role of this inflammatory transcription factor in MMT. In response to co-stimulation of TGF-β1 and IL1-β, NF-κB activation is required for E-cadherin and cytokeratin downregulation in mesothelial cells [128]. Inhibition of transforming growth factor-activated kinase-1 (TAK1) blocks the MMT changes caused by activation of NF-κB and Smad3 [141]. Moreover, the p38 MAPK signaling pathway modulates the TAK1-NF-κB pathway [142]. The MAPK kinase (MEK)-extracellular signal-regulated kinase (ERK) signaling pathway can also activate Snail expression. In this case, caveolin deficiency played a role in mesothelial cell transdifferentiation through an overactivation of MEK-ERK signaling [143].

IL-17A is known to induce phenotype changes in several cell types. In cultured human tubular-epithelial cells, these changes include loss of the epithelial marker E-cadherin and induction of a myofibroblast-like morphology in a process known as epithelial-to-mesenchymal transition (EMT), associated with proinflammatory and profibrotic factors upregulation [144]. IL-17-induced EMT promoted lung cancer cell migration and invasion via the NF-κB signaling pathway [145]. We have recently described that IL-17A also induced phenotype changes in vascular smooth muscle cells from a contractile to a synthetic cell type, leading to changes in the secretome, including upregulation of proinflammatory genes, like MCP-1 and IL-6. However, IL-17A did not increase ECM production in vascular smooth muscle cells [146]. Moreover, other studies in these cells have shown that IL-17A activates some MMT-related pathways, including ROS production, and activation of NF-κB and protein kinases, including RhoA/Rho-kinase and the MAPK cascade [30,31,137,138,139]. Interestingly, in experimental PDF exposure, the IL-17A blockade prevented the induction of MMT markers, such as α-SMA, and peritoneal fibrosis [35,108]. Thus, future studies are needed to assess whether IL-17A can directly induce MMT or can regulate this process in human mesothelial cells.

### 3.3. IL-17A in Peritoneal Fibrosis

Numerous preclinical studies have investigated the effect of IL-17A on experimental fibrosis by using different approaches, including deleting the genes of the cytokine or its receptors or blocking cytokine actions by neutralizing anti-IL-17A antibodies, but contradictory results were found. The IL-17A blockade attenuated fibrosis in some experimental models, such as lung [147,148,149], inflammatory skin [150], and intestinal fibrosis [151]. However, data of preclinical kidney damage models showed that fibrosis may be both decreased or increased by IL-17A inhibition/deletion [27,105,152,153]. Our group has demonstrated that systemic administration of IL-17A in mice for 2 weeks increased blood pressure and induced kidney inflammation but had no effect on renal collagen accumulation [61,146]. Consistent with these findings, a IL-17A neutralization treatment did not improve angiotensin II-induced experimental renal or aortic fibrosis [61]. A possible explanation could be a differential response to IL-17A in ECM production by different cell types. In fibroblast cell lines, IL-17A increased ECM synthesis [27], but this was not the case in vascular smooth muscle cells [146]. Several studies have observed that IL-17A responses can be modified in the presence of other cytokines and growth factors, showing either synergistic proinflammatory effects on endothelial cells [154] or an inhibitory effect on profibrotic responses on fibroblasts. In this respect, in human systemic sclerosis skin fibroblasts, IL-17A reduced TGF-β1-induced collagen production and α-SMA expression [155] and downregulated CTGF expression [156].

Additionally, the cell source of IL-17A and physio/pathological context may be relevant. In murine bleomycin-induced pulmonary fibrosis, IL-17A^+^/γδ^+^ T cells prevented pulmonary fibrosis, apparently through attenuation of interstitial inflammation and improving epithelial regeneration. In accordance, γδ-deficient mice exhibited increased pulmonary inflammation and ECM deposition [157]. Neutrophils are also a source of IL-17A production. At this point, it is important to highlight the possible contribution of IL-17A to ECM degradation by regulating matrix metalloproteinases (MMPs), mainly produced by neutrophils [153]. In experimental models of renal damage, IL-17 receptor knockout mice presented exacerbated renal fibrosis associated with lower neutrophil but not macrophage infiltration and diminished MMP-2 activity [153]. The authors hypothesized that IL-17 could protect against renal fibrosis by inhibiting the kallikrein-kinin system [158]. Accordingly, systemic IL-17A administration increased renal the kallikrein-1 gene and protein levels, associated with kidney neutrophil infiltration, in the absence of ECM accumulation [61]. Altogether, these data suggest the complex role of IL-17A in the regulation of ECM synthesis and degradation, and the importance of understanding the role of IL-17A in fibrosis in each individual disease. Therefore, further studies to clarify this point are needed.

Regarding peritoneum, IL-17A could contribute to peritoneal fibrosis by direct effects on resident fibroblasts. As pointed out before, peritoneal IL-17A administration to mice induced peritoneal fibrosis, characterized by fibronectin accumulation and submesothelial FSP-1 and α-SMA-stained cells [35]. The origin of these activated myofibroblasts was not evaluated in this study, but several sources have been proposed, like resident fibroblasts, phenotype conversion of mesothelial (MMT) or endothelial (EndoEMT) cells to mesenchymal cells, or infiltrating bone marrow-derived cells [159]. Importantly, in two murine models of PDF exposure, treatment with a neutralizing anti-IL-17A antibody inhibited peritoneal fibrosis; decreased the number of α-SMA expressing cells; and diminished the production of profibrotic factors such as TGF-β1, CTGF, and PAI-1 as well as extracellular matrix components, such as collagens and fibronectin [29,35]. All these processes promoted by IL-17A contribute to PM thickening and to peritoneal fibrosis progression, suggesting a potential key role of IL-17 in PM fibrosis.

### 3.4. IL-17A in Peritonitis

Peritonitis, an infection within the peritoneal cavity mainly caused by bacteria, is the most frequent complication of PD [160]. Measures to limit infection risk and to ensure prompt and appropriate investigation and treatment have lowered peritonitis rates and improved outcomes, but peritonitis remains a major determining factor in mortality and in adverse outcomes, including peritoneal inflammation and membrane failure [161]. The immune response to infectious peritonitis is initially characterized by neutrophil recruitment, with subsequent transition to monocyte predominance [162]. These changes are associated with increased intraperitoneal levels of inflammatory cytokines and neutrophil number [163]. Various studies in mice have demonstrated the importance and pleiotropy of IL-17A in peritoneal inflammatory response during infection. In one such study, abscess formation after infection or surgical injury was preceded by an increase in the number of Th17 cells in the peritoneal cavity and treatment with neutralizing antibodies against IL-17 prevented formation of the abscesses [164]. In murine peritonitis, γδ T lymphocytes are the main source of IL-17A [165,166]. Elevated intraperitoneal IL-17A levels following caecal ligation and puncture are found in mice, and intraperitoneal IL-17A blockade decreased proinflammatory cytokine production in the peritoneal cavity and caused subsequent lung injury, thus improving mouse survival [166]. In patients, the cytokine profile evident during an episode of peritonitis may predict the outcome. For example, high levels of IL-12 and IL-18 may be evident during the early phase of peritonitis and may correlate with a predominant type 1 immune response and recovery [167]. IL-17 is typically present at very low levels in PDE from uninfected patients and may increase many-fold during acute peritonitis [168]. High levels of intraperitoneal IL-17 have been correlated with favorable outcome in PD peritonitis [110]. This may suggest a protective role of IL-17A in early immune response in the peritoneal host defense but may also reflect the better outcomes seen following gram-positive bacterial infections, the class of organism where high levels of intraperitoneal IL-17 are typically seen [169].

### 3.5. IL-17A and Macrophage Functions

Macrophages play a key role in the correct function of the PM, as they modulate peritoneal inflammation and fibrosis [170,171,172]. Classically, macrophages were divided into 2 subtypes, M1 or classically activated and M2 or alternatively activated, based on cytokine expression profiles and surface markers. However, recent data suggest the existence of many mixed phenotypes depending on pathological conditions [173]. M1 macrophages produce proinflammatory factors such as IL-1β, TNF-α, IL-6, IL-23, IL-18, IL-12, and CXCL10; activate inducible nitric oxide synthase (iNOS); produce ROS; and develop cytotoxic properties [174,175,176]. M2 macrophages express indoleamine 2,3-dioxygenase, arginase I, and mannose receptor and release cytokines, like decoy IL-1RII, CCL-17, CCL-18, CCL-22, the anti-inflammatory cytokine IL-10, and profibrotic growth factors such as TGF-β1 or VEGF [177,178]. Currently, there is no clear correspondence between these human subtypes and murine macrophages due to the existence of overlapping phenotypes and different surface marker expression in different species, thus complicating the extrapolation of preclinical studies to human diseases [179]. Moreover, recent studies have increased the complexity of these classifications [173].

In PD patients, alterations of macrophage heterogeneity, characterized by different maturation and activation states, have been associated with different PD outcomes [180]. Thus, an increased proportion of the CD16^−^CD206^−^ macrophages subtype was founded in gram-negative peritonitis and failed peritonitis treatment, whereas an increased proportion of CD16^+^CD206^−^ macrophages subtype was observed in “new-starter” patients with catheter failure and stable patients with history of recurrent peritonitis episodes [180].

Peritoneal macrophages isolated from PDE of patients under continuous ambulatory PD (CAPD) during peritonitis episodes showed higher production of the proinflammatory cytokines IL-1β and TNF-α than infection-free macrophages [181]. Later studies in PDE from PD patients showed that peritoneal M2 macrophages (CD206^+^ and CD163^+^) participate in peritoneal fibrosis by favoring fibroblast overgrowth and increased CCL-18 production [182]. CCL-18 is a cytokine mainly produced by M2 macrophages associated with fibrosis/tissue repair and is increased in PDE of patients with peritonitis episodes [183,184]. Additionally, in a model of encapsulated peritoneal sclerosis, it was noted that inflammatory M2 macrophages switch to profibrotic phenotype and activate peritoneal fibroblasts through CCL-17 after sodium hypochlorite-induced injury [185]. In a model of macrophage depletion in PDF-exposed mice, transfusion of macrophages of distinct phenotypes showed a pathogenic role for M1 macrophages. M1 macrophages increased peritoneal fibrosis and disturbed peritoneal ultrafiltration more than M2 macrophages [186]. Another study of experimental PDF exposure described an increase of peritoneal thickness; fibrotic markers including collagen type I; fibronectin; and the M2 macrophage subtype markers CD206, TGF-β, Ym-1, and Arg-1. These effects were recovered by treatment with a liposome-encapsulated clodronate (LC, a specific scavenger of macrophages) [187]. A recent study demonstrated that dialyzed patients have a significantly lower content of Omega-3 fatty acids, such as n-3 Polyunsaturated fatty acid (PUFA), and this situation contributes to a high cardiovascular risk in CKD patients [188]. A study in an experimental model of PD in rats showed that the treatment with n-3 PUFA reduced peritoneal fibrosis through inhibition of activated of fibroblasts and M2 macrophages [189].

As commented before, PDF exposure models showed that IL-17A neutralization decreased submesothelial macrophage infiltration, but macrophage phenotypes and cytokine profiles were not characterized [35,108]. Another study observed that, in uremic mice, exposure to standard PDF (lactate-buffered solution) increased M1 macrophages and CD4^+^/IL-17^+^ cells in PDE [109]. In this regard, IL-17A modulates monocyte/macrophage functions such as monocyte migration, promotion of cytokine production [190,191], and macrophage phenotype modulation. In cultured macrophages derived from human THP-1 monocytes, stimulation with IL-17A increased the gene expressions of VEGF, TGF-β1, and IL-10 and upregulated M2 macrophage markers, such as CD206, CD163, Arginase I, Ym1 (also known as chitinase 3-like 3), and Fizz1 (also known as resistin-like beta) [192]. In these cells, IL-17A-induced M2 polarization was mediated through NF-κB signaling [192]. Preclinical studies confirmed the potential role of IL-17A on M1/M2 macrophage differentiation. In lung cancer cells, increased levels of IL-17A and PGE2 were involved in the development of an M2-macrophage-dominant tumor microenvironment [193]. In human and murine jawbone osteonecrosis, IL-17A mediated the M1 polarization of macrophages and serum IL-17A levels correlated with the M1/M2 macrophage ratio at the lesion foci [194]. In other diseases, such as endometriosis, IL-17A induced pathological macrophage polarization into the M2 phenotype [195]. In contrast, IL-17A-deficient mice with severe colitis presented milder intestinal inflammation and decreased M2-like macrophages, suggesting a potential beneficial effect of IL-17A in colitis [196]. In a mouse model of lipopolysaccharide (LPS)-induced peritonitis, a macrophage polarization was linked to the development and progression of infection through JAK/STAT signaling pathway [197]. Mice with *E. coli* peritonitis showed an increased IL-17A expression in immune cells including CD11b^+^ and CD11b^−^ neutrophils, macrophages, and CD3^+^ T cells [65]. In patients with cirrhosis and peritonitis, serum levels of the M2 macrophage marker CD206 were increased and associated with mortality risk [198]. In conclusion, these sometimes-controversial results require a more in-depth analysis of the specific cellular and molecular mechanisms that drive the deleterious or beneficial effect of IL-17A in macrophage polarization associated with specific pathological environments and, finally, elucidate the role of IL-17A in determining macrophage phenotype in stable PD patients or during peritonitis episodes.

### 3.6. IL-17A in Angiogenesis

One of the specific changes observed after chronic peritoneal exposure to PDF is an increased number of capillaries (angiogenesis), which is driven by VEGF and linked to an increased PM permeability [199,200,201]. In this context, mesothelial cells acquire the capacity to synthesize proinflammatory and pro-angiogenic molecules, such as VEGF [199], turning them into the main local source of VEGF during PD [125]. In cultured omentum-derived mesothelial cells, stimulation with TGFβ-1 and IL-1β to induce MMT resulted in downregulation of the two most important VEGF receptors, VEGFR1 and VEGFR2, whilst the co-receptor neuropilin-1 (Nrp1) was increased. Therefore, during in vitro MMT, the VEGF/Nrp1 interaction drives mesothelial cell behavior [202].

The potential role of IL-17A in angiogenesis induction was evaluated in proliferative disorders. The number of infiltrating IL-17A-producing cells directly correlated with microvessel density in tumors [203,204,205]. In this regard, in human colorectal carcinoma, IL-17A has been identified as an indicator of poor prognosis [205]. Accordingly, IL-17A and VEGF serum levels in patients with lung adenocarcinoma were positively correlated [206], and in tumoral cell lines, IL-17A induced VEGFA expression [205]. IL-17A-induced VEGF expression seems to be mediated by STAT3 [207,208] or STAT1 [206], but further clarification is needed concerning the molecular pathways involved and their contribution to angiogenesis. Additionally, IL-17A can also indirectly cause angiogenesis and neovascularization by stimulating the production of additional proangiogenic factors, including chemokines such as CXCL1, CXCL5, CXCL6, and CXCL8 [28,29,209]. These chemokines activate the CXCR2 receptor in endothelial cells to promote migration and proliferation [210,211]. In this regard, CXCL1 can activate VEGF signaling in gastric tumor cells [212] and CXCL8 activation of CXCR2 increased VEGF mRNA expression in cultured endothelial cells [213], suggesting a relation between proangiogenic chemokines, produced in response to IL-17A, and VEGF expression. Further studies are needed to explore the mechanisms by which IL-17A promotes VEGF expression, specifically in peritoneum exposed to PDF as well as its contribution to angiogenesis in this context (Figure 2).

## 4. Pharmacological Interference with the Th17 Immune Responses in PD

**Figure 4:**
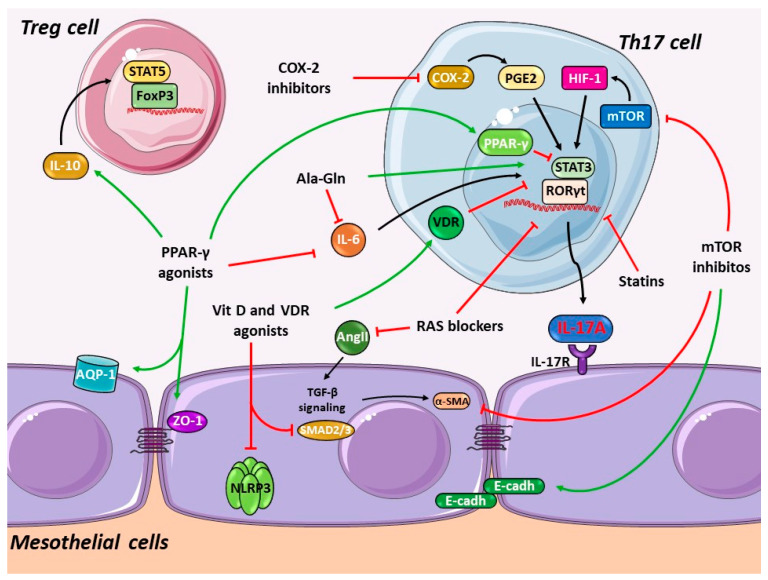
Potential therapeutic strategies modulating Th17/IL-17A response in damaged peritoneum: briefly, Peroxisome Proliferator-Activated Receptor-γ (PPAR-γ) agonists inhibit Th17 response via signal transducer and activator of transcription 3 (STAT3) blockade and downregulation of retinoid related orphan receptor γt (RORγt) and IL-6 but promote Treg response by the induction of anti-inflammatory cytokine IL-10. mTOR inhibition, via the hypoxia-induced factor-1 (HIF-1) pathway, downregulates Th17 response. Vitamin D receptor (VDR) activation and inhibition of the COX-2/PGE2 axis also target Th17 differentiation by decreased STAT3 activation and RORγt expression. Ala-Gln supplemented in PDF reduces IL-17A and IL-6 production. Other common drugs used in PD patients such as renin-angiotensin system (RAS) blockers, including angiotensin converting enzyme (ACE) inhibitors and angiotensin receptor blockers (ARBs), and statins may also modulate Th17 response. All these drugs also present other beneficial effects in the damaged peritoneum. Green arrows: activation, red arrows: inhibition.

Pharmacological therapeutic strategies that modulate T cell responses may prevent PDF-induced peritoneal damage. Those drugs could restrain Th17 differentiation or enhance Treg responses. Importantly, there is great plasticity between Th17 and Treg cells, which depends on the IL-6/TGF-*β*1 cytokine balance and specific transcription factors activation [214] and, therefore, can be modulated by drugs. Treg differentiation is controlled by TGF-*β*1, the transcription factor forkhead box P3 (FOXP3), and STAT5 activation [215]. Tregs are specialized T cells that suppress immune responses, so they maintain homeostasis and self-tolerance [216]. Treg actions are opposite to those of Th17 cells, through the secretion of anti- and proinflammatory cytokines, respectively [21,215,217,218]. Several protective pharmacological approaches with proven positive effects on PD protection can exert their beneficial effects potentially through regulation of the Th17/IL17A axis (Figure 4).

### 4.1. Blockade of the Renin-Angiotensin System

The current mainstay of CKD therapy is renin-angiotensin system (RAS) blockade using angiotensin converting enzyme (ACE) inhibitors or angiotensin receptor blockers (ARBs) [219]. These agents may be continued in PD patients to preserve residual renal function, to control blood pressure, and to decrease cardiovascular risk. RAS components are constitutively expressed in human peritoneal mesothelial cells, and peritoneal RAS is activated in response to PDF exposure, as assessed by increased generation of Angiotensin II (Ang II), the effector RAS peptide [220,221]. Ang II modulates the expression of tissue injury mediators in the peritoneum, including cytokines and chemokines leading to inflammation; growth factors such as TGF-β1, contributing to MMT of mesothelial cells; and VEGF, favoring peritoneal angiogenesis [1,221] (Figure 4). In encapsulating peritoneal sclerosis (EPS) in rats, acidic PDF exposure activated the peritoneal RAS and elicited peritoneal fibrosis, whereas the ARB olmesartan attenuated peritoneal fibrosis and peritoneal adhesions [222]. ACE inhibitors protected cultured human peritoneal mesothelial cells from high glucose-induced injury [220,223] and rats from the adverse consequences of PDF exposure [224,225]. In PD patients, ACE inhibitors appear to improve PM transport characteristics and to preserve residual kidney function [226,227,228]; however, available studies are limited. Importantly, ACE inhibitors modulate Th17 response in immune-mediated diseases. In a murine model of multiple sclerosis (experimental autoimmune encephalomyelitis, EAE), ACE inhibitors suppressed Th17 cells and induction of CD4^+^FoxP3^+^ Treg cells, together with activation of the alternative NF-κB2 pathway [229,230]. In murine obliterative airway disease, both the ACE inhibitor lisinopril and the ARB candesartan downregulated IL-17A, IL-10, and TNF-α and upregulated IL-10 via p38/MAPK pathway activation [231]. Accordingly, the ARB telmisartan also significantly decreased levels of TGF-β1, IL-17A, and TNF-α in experimental periodontitis in murine Marfan syndrome [232]. Surprisingly, in human peripheral blood mononuclear cells infected with Trypanosoma cruzi, the cause of Chagas heart disease, the ACE inhibitor captopril enhanced T. cruzi infection, decreasing the expression of the modulatory cytokine IL-10 while inducing Th17 responses [233]. However, the effects of ACE inhibitors on Th17 or Treg responses in the peritoneal milieu remain unexplored.

### 4.2. HMG-CoA Reductase Inhibitors (Statins)

Statins are lipid-lowering drugs that competitively inhibit 3-hydroxy-3-methylglutaryl-coenzyme A (HMG-CoA) reductase. In addition, they exert pleiotropic actions that could contribute to their beneficial clinical effects, as extensively demonstrated in preclinical cardiovascular disease studies [234,235]. Statins decreased inflammation-related parameters in CKD, but the cardiovascular benefits and efficacy of statins in reducing mortality rates in ERSD and dialysis patients remain uncertain [236,237,238]. Nevertheless, statins are safe in high-risk PD patients and display anti-inflammatory effects reflected in reduced serum CRP levels together with the conventional lipid-lowering effect [239]. In rat and human peritoneal mesothelial cells cultured under high glucose conditions or high glucose-PDF exposition, statins (fluvastatin, atorvastatin, or simvastatin) are protective by modulating the serum- and glucocorticoid-inducible kinase 1 (SGK1) or phospho-p38 MAPK pathways, thus inhibiting the production of growth factors and preventing MMT [240,241,242]. In rat PDF exposure models, atorvastatin preserved ultrafiltration and decreased protein loss and PM thickness [243] and simvastatin restored MMT-induced changes such as those observed in E-cadherin, α-SMA, Snail, and fibronectin expression [241]. Thus, statins may be a potential therapeutic alternative to preserve PM integrity in long-term PD patients.

Several studies have reported the immunomodulatory effects of statins. In CD4^+^ T cells isolated from multiple sclerosis patients, simvastatin augmented the production of the suppressor of cytokine secretion (SOCS) 3 and 7, negative regulators of STAT/JAK signaling, together with the induction of IFN-γ, IL-4, and IL-27, whereas they inhibited STAT1 and STAT3 activation and decreased RORγt and IL-17A mRNA levels [244,245]. In dendritic cells, atorvastatin and simvastatin lowered Th1 and/or Th17 polarization by downregulating the transcription factors T-bet and RORγt, thus inducing Treg differentiation [246]. In EAE mice, combined therapy with atorvastatin and rapamycin synergistically triggered a Treg response and attenuated Th17 cell infiltration [247]. In murine acute colitis, atorvastatin downregulated systemic cytokine levels of TNF-α, IL-17A, and the Th17-related IL-23 [248]. In nondiabetic patients undergoing PD, the addition of rosuvastatin to the ARB valsartan improved vascular dysfunction more effectively than ARB monotherapy [249]. In hypertensive patients with carotid atherosclerosis, a combination of the ARB telmisartan and rosuvastatin synergistically improved Th17/Treg functional imbalance [250] as appreciated by intensive atorvastatin treatment in patients with acute coronary syndrome [251]. Altogether, these findings show that statins may modulate Th17 and Treg responses (Figure 4); however, whether these effects occur in peritoneum during PD is unknown.

### 4.3. mTOR Inhibition

The mammalian target of rapamycin (mTOR) signaling pathway plays a key role in Th17/Treg differentiation as well as in CD8^+^ T cell and NK cell proliferation and maturation [252]. mTOR activation under hypoxic conditions accelerates Th17 differentiation of naive CD4^+^ T cells via induction of hypoxia-induced factor-1 (HIF-1) and RORγt activation with subsequent induction of IL-17A and IL-23 production [253]. In EAE mice, rapamycin, a specific mTOR inhibitor and a potent immunosuppressor, ameliorated signs of the disease by suppressing the STAT3 pathway and downregulating RORγt mRNA expression and then by reducing the number of Th17 cells and IL-17A in splenocytes [254]. Additionally, combined treatment of rapamycin and fingolimod, a sphingosine 1-phosphate receptor antagonist, prevented EAE by regulating the Akt-mTOR and MAPK/ERK pathways, which subsequently decreased IL-17A, TGF-β1, RORγt, and Foxp3, and the number of Th17 and Treg cells [255]. In Smad3-deficient mice, that are protected from TGF-β-induced peritoneal fibrosis and angiogenesis but not MMT, rapamycin lowered α-SMA expression and abrogated MMT [256]. Using this same mouse model, it has been found that HIF1α induced submesothelial thickening and angiogenesis in peritoneal tissue in a Smad3-dependent manner, whereas rapamycin blocked these effects but did not affect the direct TGFβ-mediated fibrosis and angiogenesis [257]. In cultured human peritoneal mesothelial cells, rapamycin showed a mild protective effect on MMT, increasing E-cadherin and decreasing α-SMA levels [258]. Additionally, rapamycin was protective in experimental PD, reducing PM thickness, peritoneal fibrosis, angiogenesis, lymphangiogenesis, MMT, and Endo-MT while improving peritoneal membrane transport and lipidic metabolism [259,260,261,262]. These studies suggest that mTOR inhibition by rapamycin may be an alternative to reducing IL-17A production in PD (Figure 4).

### 4.4. Cyclooxygenase-2 Inhibition

The cyclooxygenase-2 (COX-2)/prostaglandin-E2 (PGE2) pathway is a key player in PDF-induced peritoneal membrane inflammation in experimental models and patients [263]. COX-2 selective inhibition with celecoxib ameliorated ultrafiltration, fibrosis, and inflammation in both mice [263] and rat [264] models of PDF exposure. In patients, celecoxib had an anti-inflammatory effect evidenced by reduction in high-sensitivity C reactive protein (hs-CRP) levels [265]. COX-2 is implicated in promoting Th17 response, as demonstrated in vivo in COX-2 deficient mice in a model of ovalbumin-induced allergic inflammation and in vitro through COX-2 deficient CD4^+^ T cells isolated from bronchoalveolar lavage fluid of these mice [266]. COX-2 deficiency resulted in lower Th17 differentiation and decreased STAT-3 phosphorylation and RORγt expression [266]. Additionally, celecoxib significantly decreased IL-17A production in co-cultures of rheumatoid arthritis synovial fibroblasts [267]. Therefore, COX-2 inhibition appears to be a feasible therapeutic approach for peritoneal damage (Figure 4), but whether COX-2 inhibitors modulate Th17 responses in PD has not yet been explored. Nevertheless, despite the advantageous effect of COX-2 inhibition in experimental models, the clinical application of COX-2 inhibitors in PD patients must be evaluated cautiously because of the possible deleterious effect on residual renal function in these patients [268].

### 4.5. Peroxisome Proliferator-Activated Receptor-γ Agonists

Peroxisome Proliferator-Activated Receptor-γ (PPAR-γ) agonists inhibit Th17 differentiation in isolated T cells derived from multiple sclerosis and healthy patients by blocking STAT3 and downregulating RORγt, with a subsequent decrease in IL-17A protein production [269]. In preclinical studies, several PPAR-γ agonists inhibited IL-17A production. Rosiglitazone and pioglitazone downregulated mRNA and protein levels of IL-17A in lung tissue and reduced airway inflammation in murine ovalbumin-induced asthma [270]. Rosiglitazone also reduced IL-17A levels in bronchoalveolar lavage fluids from murine neutrophilic asthma [271]. Accordingly, in a murine chronic eosinophilia, rosiglitazone and 15d-PGJ(2), another PPAR-γ agonist, reduced eosinophil migration into the peritoneal cavity and decreased IL-17A and IL-23 in peritoneal exudates after 48 h of allergen challenge [272]. The first study showing that drugs could modulate Th17 and Treg responses during peritoneal damage involved PPAR-γ agonist and PDF exposure in mice [273]. Rosiglitazone decreased PDF-induced PM damage by recruiting CD3^+^ lymphocytes and CD4^+^ CD25^+^ FoxP3^+^ cells and by increasing the levels of the anti-inflammatory cytokine IL-10. Although this study suggested that the protective role of PPAR-γ agonists in the PM is mediated by Treg activation, it is worth mentioning that there was a trend towards decreased peritoneal levels of the Th17-associated cytokines IL-6 and IL-17A [273]. Further studies have also demonstrated the beneficial effects of rosiglitazone on PDF-induced injury in peritoneal cells in in vitro and in vivo models. In cultured rat peritoneal mesothelial cells, rosiglitazone decreased PDF-induced damage by inhibiting inflammation, reflected by decreased IL-6 and IL-8 production, and by preserving the mesothelial cell monolayer by regulating aquaporin-1 and zonula occludens-1 expression [274]. In later in vivo studies, rosiglitazone improved peritoneal function in rat PD with lipopolysaccharide-induced peritonitis, which was reflected in lower dialysate-to-plasma urea and albumin ratios, reduced inflammation, and a preserved mesothelial cell layer [275]. Unfortunately, neither peritoneal Th17 cell activation nor IL-17A production were assessed in these studies. Therefore, rosiglitazone emerges as a reasonable strategy to prevent peritoneal damage (Figure 4). Unfortunately, PPAR-γ agonists have a suboptimal safety profile. Thus, rosiglitazone is no longer available in the European Union due to safety concerns. Physicians prescribing pioglitazone should inform patients about potential safety concerns regarding heart failure and bladder cancer [276].

### 4.6. Alanyl-Glutamine PD Fluids Supplementation

Conventional PDF exposure induces stress responses in peritoneal cells with heat-shock proteins (HSPs) as their main effectors [277], possibly related to peritoneal and systemic glutamine depletion [278]. Glutamine is a conditionally essential amino acid for immune cell (lymphocytes, macrophages, and neutrophils) metabolism under inflammatory conditions, such as tissue damage and infection [279]. Glutamine supplementation of PDF induced HSP expression and improved PDF biocompatibility as assessed by increased mesothelial cell viability, by reduced detachment, and by decreased peritoneal protein losses in rats exposed to PDF [280]. Later, it was shown that supplementation with the stable dipeptide alanyl-glutamine (Ala-Gln) of heat-sterilized glucose-based PDF restored peritoneal cell stress responses in both the immortalized cell line MeT-5A and in primary cultures of human mesothelial cells from uremic patients [278]. In PD patients, Ala-Gln supplementation improved HSP-mediated stress response but did not modify PDE levels of inflammatory cytokines (IL-8, IL-6, or TNF-α). However, in a subgroup of patients with previous history of peritonitis, Ala-Gln supplementation attenuated IL-8 but no IL-6 levels in PDE [281]. In this context, normal human peripheral blood mononuclear cells (PBMCs) ex vivo exposed to PDE from PD patients treated with Ala-Gln-supplemented PDF showed higher LPS-stimulated TNF-α release than when stimulated with PDE from conventional PDF-treated patients, suggesting an improved peritoneal host-defense [281]. Additionally, in murine PD-related peritonitis, Aln-Gln reduced TNF-α and IL-6 PDE levels, whereas it increased and restored the ex vivo release of these cytokines in LPS-stimulated PDE peritoneal cells [281]. Similar results were observed in ex vivo LPS-stimulated human PDE cells [282]. Transcriptomic analysis of PDE cells from PD patients treated with and without Ala-Gln-supplemented PDF identified as highest-ranked networks the downregulated TNF-α transcript and Akt and IL-1R signaling, whereas pathway analysis identified IL-6 signaling-related pathways as the most significantly enriched [282]. Interestingly, in rat PDF exposure, Ala-Gln supplementation attenuated PM thickness, α-SMA expression, and angiogenesis (CD31^+^ vessels), together with impaired Th17 responses, reflected by reduced IL-17A, TGF-β1, IL-6, and RORγt peritoneal levels [283]. Thus, addition of Ala-Gln to PDF emerges as a good strategy to reduce peritoneal injury and inflammation and to improve immune responses in PD patients by pathways that could involve Th17 modulation (Figure 4).

### 4.7. Vitamin D and Related Drugs

Paricalcitol, an analog of vitamin D and a specific activator of vitamin D receptors (VDR), reduced PDF-induced peritoneal fibrosis in murine PD and decreased peritoneal IL-17A levels but not of other cytokines, including IL-1β, IL-2, IL-4, IL-5, IL-6, IL-10, TNF-α, IFN-γ, and TGF-β1. These beneficial effects were associated with increased numbers of CD4^+^ and CD8^+^ Tregs, suggesting that protective actions of paricalcitol could be related to Treg activation [284]. Later studies confirmed that paricalcitol fully prevents loss of ultrafiltration capacity induced by PDF exposure in rats and decreased PM thickening and angiogenesis [285]. Paricalcitol also exerted other beneficial effects, like attenuation of in vitro MMT through Smads modulation [286]; impairment of oxidative stress; and activation of the NLRP3 inflammasome, a multiprotein complex that promotes IL-1β and IL-18 maturation [287]. Interestingly, a recent retrospective study in prevalent PD patients observed lower peritoneal protein loss in patients treated with paricalcitol [288]. Thus, paricalcitol acts as an anti-inflammatory agent that might improve peritoneal alterations in PD by mechanisms that could involve IL-17A modulation (Figure 4).

## 5. MicroRNAs in Th17 and PD

MicroRNAs (miRNAs) are emerging as potential biomarkers and/or therapeutic targets in multiple conditions, including kidney disease [289]. They are a large family of conserved, small (about 22 nucleotides), noncoding, and single-stranded RNAs that repress the translation and/or induce the degradation of their mRNA targets [290]. In PD patients, miRNA deregulation has been described in PDE, the peritoneal cavity, and serum. In PDE, miR-21 and miR-21-5p were increased whereas miR-129-5p, miR-200c, and miR-589 were decreased [291]. In peritoneal tissue, miR-21, miR-23, miR-199a-5p, and miR-214-3p were upregulated and miR-30a was downregulated. In line with these findings, in the same PD patients, serum levels of miR-21-5p, miR-221-3p, and miR-327 were elevated and miR-34a-5p was decreased [291]. In another study, peritoneal transport characteristics were associated with miR-15a, miR-17, miR-21, miR-30, miR-192, and miR-377 levels [292]. Regarding potential miRNAs involved in Th17/IL-17A pathway regulation, most studies have been carried out in autoimmune diseases [98,293]. Curiously, in an in silico study, some of the miRNAs deregulated in PD patients (e.g., miR-17, miR-21, miR-129-5p, miR-377, or miR-589) were proposed to interact with the mRNAs of Th17-related cytokines and IL-17 receptors [294]. Of these predicted miRNAs, miR-21 could regulate Th17 immune responses as it promotes Th17 differentiation in experimental EAE [295]. A correlation between miR-21 levels and the Treg/Th17 ratio was found in experimental rat hepatocellular carcinoma [296]. In patients with rheumatoid arthritis, decreased miR-21 expression correlated with an imbalance of Th-17 and Treg cells [297]. Further studies would be necessary to evaluate the role of these miRNAs, in particular miR-21, in the regulation of Th17 responses during peritoneal damage secondary to PDF and/or peritonitis.

## 6. Beyond the Peritoneum

The daily repeated infusion of current PDF into the peritoneal cavity may elicit an array of local and systemic untoward effects, including PM damage, sterile inflammation, and peritoneal vascular reactivity [200], as well as systemic metabolic, inflammatory and immune modulatory effects in which the impact on patient outcomes has not yet been properly characterized. It is well known that PD patients, besides renal dysfunction, frequently present other complications, including hypertension, diabetes, and cardiovascular pathologies. Importantly, around 40–60% of deaths in PD patients are due to cardiovascular events [298,299,300]. Of interest, IL-17A contributes to the pathogenesis of several cardiovascular disorders, mainly hypertension, atherosclerosis, and ischemic heart disease [301,302,303,304,305]. Thus, immune and nonimmune kidney diseases, cardiovascular complications, and peritoneal damage in PD patients must be considered in the treatment of these patients. The data reviewed here also suggest a potential role of IL-17A in the cardio–renal axis, and future research in this context is needed.

## 7. Clinical Targeting of IL-17A

From a clinical translation point of view, several ongoing clinical trials (https://clinicaltrials.gov/) are testing anti-IL-17A neutralizing antibodies for chronic inflammatory diseases [306,307,308], such as Crohn’s disease (NCT00936585), spondyloarthritis (NCT03358134), and psoriasis (NCT01892436). Hence, IL-17A blockade by neutralizing antibodies has already reached the clinical development stage, thus facilitating potential future trials aimed at addressing the role of IL-17A blockade as a new therapeutic approach to attenuate peritoneal damage induced by chronic PDF exposure in PD patients.

## 8. Conclusions

In conclusion, the data reviewed here suggest a potential role of IL-17A in peritoneal membrane injury during PD as well as in the cardio-renal axis that induces poor PD patient outcomes. Key issues to be addressed are the identification of the main drivers of IL17A responses in clinical PD and the need for and design of clinical trials targeting IL17A in PD.

Future research should unravel the impact of PDF biocompatibility on recruitment of IL17A responses in PD patients. Preclinical studies suggested that novel biocompatible PDF did not activate Th17 immune responses [109]. However, there is scarce clinical information on whether this is the case in PD patients. Detailed studies are needed on IL-17A levels in peritoneal effluent from PD patients treated with different PDF as well as analysis of peritoneal biopsies for activation of IL17A responses in relation to the prior or current use of less biocompatible PDF. These studies could suggest whether deleterious IL-17A responses could be controlled by an optimized choice of PDF. Even new enriched PDF should be tested in this respect.

If evidence is found of continuing activation of deleterious IL17A responses even in patients using biocompatible PDF, the next step may be the design of a pilot clinical trial using specific anti-IL17A strategies. The nature of PD allows for the design of studies using local intraperitoneal administration of IL-17A-taregting drugs to minimize systemic exposure and adverse effects as well as cost. Endpoints to be considered for these future trials include peritoneal (peritoneal function, peritonitis episodes, and biopsies), renal (residual renal function), and cardiovascular (events) endpoints. We recognize that it is currently unknown whether the local control of IL-17A responses at the peritoneum may have a beneficial impact on kidney and cardiovascular outcomes by controlling a source of systemic inflammation. Preclinical studies may be helpful in assessing whether local intraperitoneal or systemic delivery of IL-17A-targeting therapies is preferable.

## References

[1] Ruiz-Ortega M., Rayego-Mateos S., Lamas S., Ortiz A., Rodrigues-Diez R.R. (2020). Targeting the progression of chronic kidney disease. Nat. Rev. Nephrol..

[2] del Peso G., Jiménez-Heffernan J.A., Selgas R., Remón C., Ossorio M., Fernández-Perpén A., Sánchez-Tomero J.A., Cirugeda A., de Sousa E., Sandoval P. (2016). Biocompatible dialysis solutions preserve peritoneal mesothelial cell and vessel wall integrity. A case-control study on human biopsies. Perit. Dial. Int..

[3] Zhou Q., Bajo M.A., del Peso G., Yu X., Selgas R. (2016). Preventing peritoneal membrane fibrosis in peritoneal dialysis patients. Kidney Int..

[4] Mortier S., De Vriese A.S., Van de Voorde J., Schaub T.P., Passlick-Deetjen J., Lameire N.H. (2002). Hemodynamic Effects of Peritoneal Dialysis Solutions on the Rat Peritoneal Membrane: Role of Acidity, Buffer Choice, Glucose Concentration, and Glucose Degradation Products. J. Am. Soc. Nephrol..

[5] Jörres A. (2012). Novel peritoneal dialysis solutions-What are the clinical implications?. Blood Purif..

[6] Ortiz A., Wieslander A., Linden T., Santamaria B., Sanz A., Justo P., Sanchez-Nino M.-D., Benito A., Kjellstrand P. (2006). 3,4-DGE is Important for Side Effects in Peritoneal Dialysis What About its Role in Diabetes. Curr. Med. Chem..

[7] Catalan M.P., Santamaría B., Reyero A., Ortiz A., Egido J., Ortiz A. (2005). 3,4-Di-deoxyglucosone-3-ene promotes leukocyte apoptosis. Kidney Int..

[8] Santamaría B., Ucero A.C., Benito-Martin A., Vicent M.J., Orzáez M., Celdrán A., Selgas R., Ruíz-Ortega M., Ortiz A. (2015). Biocompatibility reduces inflammation-induced apoptosis in mesothelial cells exposed to peritoneal dialysis Fluid. Blood Purif..

[9] Baroni G., Schuinski A., de Moraes T.P., Meyer F., Pecoits-Filho R. (2012). Inflammation and the Peritoneal Membrane: Causes and Impact on Structure and Function during Peritoneal Dialysis. Mediat. Inflamm..

[10] Velloso M.S.S., Otoni A., de Paula Sabino A., de Castro W.V., Pinto S.W.L., Marinho M.A.S., Rios D.R.A. (2014). Peritoneal dialysis and inflammation. Clin. Chim. Acta.

[11] Devuyst O., Margetts P.J., Topley N. (2010). The pathophysiology of the peritoneal membrane. J. Am. Soc. Nephrol..

[12] Lee C.T., Ng H.Y., Hsu C.Y., Tsai Y.C., Yang Y.K., Chen T.C., Chiou T.T.Y., Kuo C.C., Lee W.C., Hsu K.T. (2010). Proinflammatory cytokines, hepatocyte growth factor and adipokines in peritoneal dialysis patients. Artif. Organs.

[13] Sawai A., Ito Y., Mizuno M., Suzuki Y., Toda S., Ito I., Hattori R., Matsukawa Y., Gotoh M., Takei Y. (2010). Peritoneal macrophage infiltration is correlated with baseline peritoneal solute transport rate in peritoneal dialysis patients. Nephrol. Dial. Transplant..

[14] Parikova A., Zweers M.M., Struijk D.G., Krediet R.T. (2003). Peritoneal effluent markers of inflammation in patients treated with icodextrin-based and glucose-based dialysis solutions. Adv. Perit. Dial..

[15] Sanz A.B., Aroeira L.S., Bellon T., Del Peso G., Jimenez-Heffernan J., Santamaria B., Sanchez-Niño M.D., Blanco-Colio L.M., Lopez-Cabrera M., Ruiz-Ortega M. (2014). TWEAK promotes peritoneal inflammation. PLoS ONE.

[16] Schaefer B., Bartosova M., Macher-Goeppinger S., Sallay P., Vörös P., Ranchin B., Vondrak K., Ariceta G., Zaloszyc A., Bayazit A.K. (2018). Neutral pH and low–glucose degradation product dialysis fluids induce major early alterations of the peritoneal membrane in children on peritoneal dialysis. Kidney Int..

[17] Lui S.L., Yung S., Yim A., Wong K.M., Tong K.L., Wong K.S., Li C.S., Au T.C., Lo W.K., Ho Y.W. (2012). A combination of biocompatible peritoneal dialysis solutions and residual renal function, peritoneal transport, and inflammation markers: A randomized clinical trial. Am. J. Kidney Dis..

[18] Schmidt T., Luebbe J., Paust H.J., Panzer U. (2018). Mechanisms and functions of IL-17 signaling in renal autoimmune diseases. Mol. Immunol..

[19] Wang E.A., Suzuki E., Maverakis E., Adamopoulos I.E. (2017). Targeting IL-17 in psoriatic arthritis. Eur. J. Rheumatol..

[20] Jin W., Dong C. (2013). IL-17 cytokines in immunity and inflammation. Emerg. Microbes Infect..

[21] Beringer A., Noack M., Miossec P. (2016). IL-17 in Chronic Inflammation: From Discovery to Targeting. Trends Mol. Med..

[22] Nordlohne J., von Vietinghoff S. (2019). Interleukin 17A in atherosclerosis—Regulation and pathophysiologic effector function. Cytokine.

[23] Gong F., Liu Z., Liu J., Zhou P., Liu Y., Lu X. (2015). The paradoxical role of IL-17 in atherosclerosis. Cell. Immunol..

[24] Aggarwal S., Gurney A.L. (2002). IL-17: Prototype member of an emerging cytokine family. J. Leukoc. Biol..

[25] von Vietinghoff S., Ley K. (2010). Interleukin 17 in vascular inflammation. Cytokine Growth Factor Rev..

[26] Krstic J., Obradovic H., Kukolj T., Mojsilovic S., Okic-Dordevic I., Bugarski D., Santibanez J. (2015). An Overview of Interleukin-17A and Interleukin-17 Receptor A Structure, Interaction and Signaling. Protein Pept. Lett..

[27] Ramani K., Biswas P.S. (2019). Interleukin-17: Friend or foe in organ fibrosis. Cytokine.

[28] Wu L., Awaji M., Saxena S., Varney M.L., Sharma B., Singh R.K. (2020). IL-17–CXC Chemokine Receptor 2 Axis Facilitates Breast Cancer Progression by Up-Regulating Neutrophil Recruitment. Am. J. Pathol..

[29] Witowski J., Kamhieh-Milz J., Kawka E., Catar R., Jörres A. (2018). IL-17 in Peritoneal Dialysis-Associated Inflammation and Angiogenesis: Conclusions and Perspectives. Front. Physiol..

[30] Zhang H., Chen J., Liu X., Awar L., Mcmickle A., Bai F., Nagarajan S., Yu S. (2013). IL-17 induces expression of vascular cell adhesion molecule through signalling pathway of NF-κB, but not Akt1 and TAK1 in vascular smooth muscle cells. Scand. J. Immunol..

[31] Pietrowski E., Bender B., Huppert J., White R., Luhmann H.J., Kuhlmann C.R.W. (2010). Pro-inflammatory effects of interleukin-17A on vascular smooth muscle cells involve NAD(P)H- oxidase derived reactive oxygen species. J. Vasc. Res..

[32] Gu C., Wu L., Li X. (2013). IL-17 family: Cytokines, receptors and signaling. Cytokine.

[33] Blauvelt A., Chiricozzi A. (2018). The Immunologic Role of IL-17 in Psoriasis and Psoriatic Arthritis Pathogenesis. Clin. Rev. Allergy Immunol..

[34] Cua D.J., Tato C.M. (2010). Innate IL-17-producing cells: The sentinels of the immune system. Nat. Rev. Immunol..

[35] Rodrigues-Díez R., Aroeira L.S., Orejudo M., Bajo M.A., Heffernan J.J., Rodrigues-Díez R.R., Rayego-Mateos S., Ortiz A., Gonzalez-Mateo G., López-Cabrera M. (2014). IL-17A is a novel player in dialysis-induced peritoneal damage. Kidney Int..

[36] Gagliani N., Huber S. (2017). Basic aspects of T helper cell differentiation. Methods in Molecular Biology.

[37] Saigusa R., Winkels H., Ley K. (2020). T cell subsets and functions in atherosclerosis. Nat. Rev. Cardiol..

[38] Cosmi L., Maggi L., Santarlasci V., Liotta F., Annunziato F. (2014). T helper cells plasticity in inflammation. Cytom. Part A.

[39] Raphael I., Nalawade S., Eagar T.N., Forsthuber T.G. (2015). T cell subsets and their signature cytokines in autoimmune and inflammatory diseases. Cytokine.

[40] Mesquita D., Kirsztajn G.M., Franco M.F., Reis L.A., Perazzio S.F., Mesquita F.V., da Silva Ferreira V., Andrade L.E.C., de Souza A.W.S. (2018). CD4^+^ T helper cells and regulatory T cells in active lupus nephritis: An imbalance towards a predominant Th1 response?. Clin. Exp. Immunol..

[41] Waite J.C., Skokos D. (2012). Th17 Response and Inflammatory Autoimmune Diseases. Int. J. Inflamm..

[42] Romagnani S. (1992). Type 1 T helper and type 2 T helper cells: Functions, regulation and role in protection and disease. Int. J. Clin. Lab. Res..

[43] Dupage M., Bluestone J.A. (2016). Harnessing the plasticity of CD4+ T cells to treat immune-mediated disease. Nat. Rev. Immunol..

[44] Ivanov I.I., McKenzie B.S., Zhou L., Tadokoro C.E., Lepelley A., Lafaille J.J., Cua D.J., Littman D.R. (2006). The Orphan Nuclear Receptor RORγt Directs the Differentiation Program of Proinflammatory IL-17^+^ T Helper Cells. Cell.

[45] Nanda A.S., Ward W.R., Dobson H. (1991). Lack of LH response to oestradiol treatment in cows with cystic ovarian disease and effect of progesterone treatment or manual rupture. Res. Vet. Sci..

[46] Volpe E., Servant N., Zollinger R., Bogiatzi S.I., Hupé P., Barillot E., Soumelis V. (2008). A critical function for transforming growth factor-β, interleukin 23 and proinflammatory cytokines in driving and modulating human TH-17 responses. Nat. Immunol..

[47] Capone A., Volpe E. (2020). Transcriptional Regulators of T Helper 17 Cell Differentiation in Health and Autoimmune Diseases. Front. Immunol..

[48] Harris T.J., Grosso J.F., Yen H.-R., Xin H., Kortylewski M., Albesiano E., Hipkiss E.L., Getnet D., Goldberg M.V., Maris C.H. (2007). Cutting Edge: An In Vivo Requirement for STAT3 Signaling in T H 17 Development and T H 17-Dependent Autoimmunity. J. Immunol..

[49] Robert M., Miossec P. (2017). Effects of Interleukin 17 on the cardiovascular system. Autoimmun. Rev..

[50] Yasuda K., Takeuchi Y., Hirota K. (2019). The pathogenicity of Th17 cells in autoimmune diseases. Semin. Immunopathol..

[51] Rodriguez-Iturbe B., Pons H., Johnson R.J. (2017). Role of the immune system in hypertension. Physiol. Rev..

[52] Patel D.D., Kuchroo V.K. (2015). Th17 Cell Pathway in Human Immunity: Lessons from Genetics and Therapeutic Interventions. Immunity.

[53] Liappas G., Gónzalez-Mateo G.T., Majano P., Sánchez-Tomero J.A., Ruiz-Ortega M., Rodrigues Díez R., Martín P., Sanchez-Díaz R., Selgas R., López-Cabrera M. (2015). T Helper 17/Regulatory T Cell Balance and Experimental Models of Peritoneal Dialysis-Induced Damage. Biomed Res. Int..

[54] Boldizsar F., Tarjanyi O., Nemeth P., Mikecz K., Glant T.T. (2009). Th1/Th17 polarization and acquisition of an arthritogenic phenotype in arthritis-susceptible BALB/c, but not in MHC-matched, arthritis-resistant DBA/2 mice. Int. Immunol..

[55] Hou Y.C., Liu J.J., Pai M.H., Tsou S.S., Yeh S.L. (2013). Alanyl-glutamine administration suppresses Th17 and reduces inflammatory reaction in dextran sulfate sodium-induced acute colitis. Int. Immunopharmacol..

[56] Shiromizu C.M., Jancic C.C. (2018). γδ T lymphocytes: An effector cell in autoimmunity and infection. Front. Immunol..

[57] Fenoglio D., Poggi A., Catellani S., Battaglia F., Ferrera A., Setti M., Murdaca G., Zocchi M.R. (2009). Vδ1 T lymphocytes producing IFN-γ and IL-17 are expanded in HIV-1-infected patients and respond to *Candida albicans*. Blood.

[58] Hirata T., Osuga Y., Takamura M., Saito A., Hasegawa A., Koga K., Yoshino O., Hirota Y., Harada M., Taketani Y. (2011). Interleukin-17F increases the secretion of interleukin-8 and the expression of cyclooxygenase 2 in endometriosis. Fertil. Steril..

[59] Li Y., Wu Y., Zhang C., Li P., Cui W., Hao J., Ma X., Yin Z., Du J. (2014). γδT cell-derived interleukin-17A via an interleukin-1β- dependent mechanism mediates cardiac Injury and Fibrosis in hypertension. Hypertension.

[60] Saleh M.A., Norlander A.E., Madhur M.S. (2016). Inhibition of Interleukin-17A, But Not Interleukin-17F, Signaling Lowers Blood Pressure, and Reduces End-Organ Inflammation in Angiotensin II–Induced Hypertension. JACC Basic to Transl. Sci..

[61] Orejudo M., Rodrigues-Diez R.R., Rodrigues-Diez R., Garcia-Redondo A., Santos-Sánchez L., Rández-Garbayo J., Cannata-Ortiz P., Ramos A.M., Ortiz A., Selgas R. (2019). Interleukin 17A participates in renal inflammation associated to experimental and human hypertension. Front. Pharmacol..

[62] Rei M., Goncąlves-Sousa N., Lancą T., Thompson R.G., Mensurado S., Balkwill F.R., Kulbe H., Pennington D.J., Silva-Santos B. (2014). Murine CD27(-) Vγ6(+) γδ T cells producing IL-17A promote ovarian cancer growth via mobilization of protumor small peritoneal macrophages. Proc. Natl. Acad. Sci. USA.

[63] Guo Y., Sun X., Shibata K., Yamada H., Muta H., Podack E.R., Yoshikai Y. (2013). CD30 is required for activation of a unique subset of interleukin- 17A-Producing γδT Cells in innate immunity against mycobacterium bovis bacillus calmette-guérin infection. Infect. Immun..

[64] Shibata K., Yamada H., Hara H., Kishihara K., Yoshikai Y. (2007). Resident Vδ1 + γδ T Cells Control Early Infiltration of Neutrophils after Escherichia coli Infection via IL-17 Production. J. Immunol..

[65] Ren Y., Hua L., Meng X., Xiao Y., Hao X., Guo S., Zhao P., Wang L., Dong B., Yu Y. (2016). Correlation of Surface Toll-Like Receptor 9 Expression with IL-17 Production in Neutrophils during Septic Peritonitis in Mice Induced by *E. coli*. Mediat. Inflamm..

[66] Lin A.M., Rubin C.J., Khandpur R., Wang J.Y., Riblett M., Yalavarthi S., Villanueva E.C., Shah P., Kaplan M.J., Bruce A.T. (2011). Mast Cells and Neutrophils Release IL-17 through Extracellular Trap Formation in Psoriasis. J. Immunol..

[67] Li R.H.L., Tablin F. (2018). A comparative review of neutrophil extracellular traps in sepsis. Front. Vet. Sci..

[68] Pelletier M., Maggi L., Micheletti A., Lazzeri E., Tamassia N., Costantini C., Cosmi L., Lunardi C., Annunziato F., Romagnani S. (2010). Evidence for a cross-talk between human neutrophils and Th17 cells. Blood.

[69] Krystel-Whittemore M., Dileepan K.N., Wood J.G. (2016). Mast cell: A multi-functional master cell. Front. Immunol..

[70] Hueber A.J., Asquith D.L., Miller A.M., Reilly J., Kerr S., Leipe J., Melendez A.J., McInnes I.B. (2010). Mast cells express IL-17A in rheumatoid arthritis synovium. J. Immunol..

[71] Liu X., Jin H., Zhang G., Lin X., Chen C., Sun J., Zhang Y., Zhang Q., Yu J. (2014). Intratumor IL-17-positive mast cells are the major source of the IL-17 that is predictive of survival in gastric cancer patients. PLoS ONE.

[72] Kenna T.J., Brown M.A. (2013). The role of IL-17-secreting mast cells in inflammatory joint disease. Nat. Rev. Rheumatol..

[73] Noordenbos T., Blijdorp I., Chen S., Stap J., Mul E., Cañete J.D., Lubberts E., Yeremenko N., Baeten D. (2016). Human mast cells capture, store, and release bioactive, exogenous IL-17A. J. Leukoc. Biol..

[74] Jiménez-Heffernan J.A., Bajo M.A., Perna C., Del Peso G., Larrubia J.R., Gamallo C., Sánchez-Tomero J.A., López-Cabrera M., Selgas R. (2006). Mast cell quantification in normal peritoneum and during peritoneal dialysis treatment. Arch. Pathol. Lab. Med..

[75] Zareie M., Fabbrini P., Hekking L.H.P., Keuning E.D., Ter Wee P.M., Beelen R.H.J., Van Den Born J. (2006). Novel role for mast cells in omental tissue remodeling and cell recruitment in experimental peritoneal dialysis. J. Am. Soc. Nephrol..

[76] Rönnberg E., Johnzon C.F., Calounova G., Garcia Faroldi G., Grujic M., Hartmann K., Roers A., Guss B., Lundequist A., Pejler G. (2014). Mast cells are activated by Staphylococcus aureus in vitro but do not influence the outcome of intraperitoneal S. aureus infection in vivo. Immunology.

[77] Dahdah A., Gautier G., Attout T., Fiore F., Lebourdais E., Msallam R., Daëron M., Monteiro R.C., Benhamou M., Charles N. (2014). Mast cells aggravate sepsis by inhibiting peritoneal macrophage phagocytosis. J. Clin. Investig..

[78] Bradding P., Pejler G. (2018). The controversial role of mast cells in fibrosis. Immunol. Rev..

[79] Zareie M., Hekking L.H., Driesprong B.A., ter Wee P.M., Beelen R.H., van den Born J. (2001). Accumulation of omental mast cells during peritoneal dialysis. Perit. Dial. Int..

[80] Alscher D.M., Braun N., Biegger D., Fritz P. (2007). Peritoneal Mast Cells in Peritoneal Dialysis Patients, Particularly in Encapsulating Peritoneal Sclerosis Patients. Am. J. Kidney Dis..

[81] Kazama I., Baba A., Endo Y., Toyama H., Ejima Y., Matsubara M., Tachi M. (2015). Mast cell involvement in the progression of peritoneal fibrosis in rats with chronic renal failure. Nephrology.

[82] Dusseaux M., Martin E., Serriari N., Péguillet I., Premel V., Louis D., Milder M., Le Bourhis L., Soudais C., Treiner E. (2011). Human MAIT cells are xenobiotic-resistant, tissue-targeted, CD161 hi IL-17-secreting T cells. Blood.

[83] Kjer-Nielsen L., Patel O., Corbett A.J., Le Nours J., Meehan B., Liu L., Bhati M., Chen Z., Kostenko L., Reantragoon R. (2012). MR1 presents microbial vitamin B metabolites to MAIT cells. Nature.

[84] Reantragoon R., Corbett A.J., Sakala I.G., Gherardin N.A., Furness J.B., Chen Z., Eckle S.B.G., Uldrich A.P., Birkinshaw R.W., Patel O. (2013). Antigen-loaded MR1 tetramers define T cell receptor heterogeneity in mucosal-associated invariant T cells. J. Exp. Med..

[85] Ibidapo-obe O., Stengel S., Köse-Vogel N., Quickert S., Reuken P.A., Busch M., Bauer M., Stallmach A., Bruns T. (2020). Mucosal-Associated Invariant T Cells Redistribute to the Peritoneal Cavity During Spontaneous Bacterial Peritonitis and Contribute to Peritoneal Inflammation. Cell. Mol. Gastroenterol. Hepatol..

[86] Summers S.A., Steinmetz O.M., Li M., Kausman J.Y., Semple T., Edgtton K.L., Borza D.B., Braley H., Holdsworth S.R., Kitching A.R. (2009). Th1 and Th17 cells induce proliferative glomerulonephritis. J. Am. Soc. Nephrol..

[87] Ghali J., Holdsworth S., Kitching A. (2015). Targeting IL-17 and IL-23 in Immune Mediated Renal Disease. Curr. Med. Chem..

[88] Dolff S., Witzke O., Wilde B. (2019). Th17 cells in renal inflammation and autoimmunity. Autoimmun. Rev..

[89] Nogueira E., Hamour S., Sawant D., Henderson S., Mansfield N., Chavele K.-M., Pusey C.D., Salama A.D. (2010). Serum IL-17 and IL-23 levels and autoantigen-specific Th17 cells are elevated in patients with ANCA-associated vasculitis. Nephrol. Dial. Transplant..

[90] Krohn S., Nies J.F., Kapffer S., Schmidt T., Riedel J.H., Kaffke A., Peters A., Borchers A., Steinmetz O.M., Krebs C.F. (2018). IL-17C/IL-17 receptor E signaling in CD4^+^ T cells promotes T H 17 cell-driven glomerular inflammation. J. Am. Soc. Nephrol..

[91] Kwan B.C.-H., Tam L.-S., Lai K.-B., Lai F.M.-M., Li E.K.-M., Wang G., Chow K.-M., Li P.K.-T., Szeto C.-C. (2009). The gene expression of type 17 T-helper cell-related cytokines in the urinary sediment of patients with systemic lupus erythematosus. Rheumatology (Oxford).

[92] de Oliveira Peliçari K., Postal M., Sinicato N.A., Peres F.A., Fernandes P.T., Marini R., Costallat L.T.L., Appenzeller S. (2015). Serum interleukin-17 levels are associated with nephritis in childhood-onset systemic lupus erythematosus. Clinics (Sao Paulo).

[93] Kalavrizioti D., Gerolymos M., Rodi M., Kalliakmani P., Provatopoulou S., Eleftheriadis T., Mouzaki A., Goumenos D.S. (2015). T helper (Th)-cytokines in the urine of patients with primary glomerulonephritis treated with immunosuppressive drugs: Can they predict outcome?. Cytokine.

[94] Ramani K., Biswas P.S. (2016). Emerging roles of the Th17/IL-17-axis in glomerulonephritis. Cytokine.

[95] Krebs C.F., Lange S., Niemann G., Rosendahl A., Lehners A., Meyer-Schwesinger C., Stahl R.A.K., Benndorf R.A., Velden J., Paust H.J. (2014). Deficiency of the interleukin 17/23 axis accelerates renal injury in mice with deoxycorticosterone acetate+angiotensin II-induced hypertension. Hypertension.

[96] Lavoz C., Matus Y.S., Orejudo M., Carpio J.D., Droguett A., Egido J., Mezzano S., Ruiz-Ortega M. (2019). Interleukin-17A blockade reduces albuminuria and kidney injury in an accelerated model of diabetic nephropathy. Kidney Int..

[97] Ma J., Li Y.J., Chen X., Kwan T., Chadban S.J., Wu H. (2019). Interleukin 17A promotes diabetic kidney injury. Sci. Rep..

[98] Lavoz C., Rayego-Mateos S., Orejudo M., Opazo-Ríos L., Marchant V., Marquez-Exposito L., Tejera-Muñoz A., Navarro-González J.F., Droguett A., Ortiz A. (2020). Could IL-17A Be a Novel Therapeutic Target in Diabetic Nephropathy?. J. Clin. Med..

[99] Niewczas M.A., Pavkov M.E., Skupien J., Smiles A., Md Dom Z.I., Wilson J.M., Park J., Nair V., Schlafly A., Saulnier P.J. (2019). A signature of circulating inflammatory proteins and development of end-stage renal disease in diabetes. Nat. Med..

[100] Li P.K.T., Ng J.K.C., Mcintyre C.W. (2017). Inflammation and Peritoneal Dialysis. Semin. Nephrol..

[101] Zamauskaite A., Yaqoob M.M., Madrigal J.A., Cohen S.B. (1999). The frequency of Th2 type cells increases with time on peritoneal dialysis in patients with diabetic nephropathy. Eur. Cytokine Netw..

[102] Zhu X., Li S., Zhang Q., Zhu D., Xu Y., Zhang P., Han J., Duan Z., Gao J., Ou Y. (2018). Correlation of increased Th17/Treg cell ratio with endoplasmic reticulum stress in chronic kidney disease. Medicine (United States).

[103] Ma L., Zhang H., Hu K., Lv G., Fu Y., Ayana D.A., Zhao P., Jiang Y. (2015). The imbalance between Tregs, Th17 cells and inflammatory cytokines among renal transplant recipients. BMC Immunol..

[104] Romanova Y., Laikov A., Markelova M., Khadiullina R., Makseev A., Hasanova M., Rizvanov A., Khaiboullina S., Salafutdinov I. (2020). Proteomic analysis of human serum from patients with chronic kidney disease. Biomolecules.

[105] Sun B., Wang H., Zhang L., Yang X., Zhang M., Zhu X., Ji X., Wang H. (2018). Role of interleukin 17 in TGF-β signaling-mediated renal interstitial fibrosis. Cytokine.

[106] Rodrigues-Díez R., Rodrigues-Díez R.R., Rayego-Mateos S., Suarez-Alvarez B., Lavoz C., Stark Aroeira L., Sánchez-López E., Orejudo M., Alique M., Lopez-Larrea C. (2013). The C-terminal module IV of connective tissue growth factor is a novel immune modulator of the Th17 response. Lab. Investig..

[107] Rosendahl A., Kabiri R., Bode M., Cai A., Klinge S., Ehmke H., Mittrücker H.W., Wenzel U.O. (2019). Adaptive immunity and IL-17A are not involved in the progression of chronic kidney disease after 5/6 nephrectomy in mice. Br. J. Pharmacol..

[108] Liappas G., González-Mateo G.T., Sánchez-Díaz R., Lazcano J.J., Lasarte S., Matesanz-Marín A., Zur R., Ferrantelli E., Ramírez L.G., Aguilera A. (2016). Immune-regulatory molecule CD69 controls peritoneal fibrosis. J. Am. Soc. Nephrol..

[109] Vila Cuenca M., Keuning E.D., Talhout W., Paauw N.J., van Ittersum F.J., ter Wee P.M., Beelen R.H.J., Vervloet M.G., Ferrantelli E. (2018). Differences in peritoneal response after exposure to low-GDP bicarbonate/lactate-buffered dialysis solution compared to conventional dialysis solution in a uremic mouse model. Int. Urol. Nephrol..

[110] Wang H.H., Lee T.Y., Lin C.Y. (2011). Kinetics and involvement of interleukin-17 in the outcome of peritonitis in nondiabetic patients undergoing peritoneal dialysis. J. Chin. Med. Assoc..

[111] Gonzalez-Mateo G.T., Loureiro J., Jimenez-Hefferman J.A., Bajo M.-A., Selgas R., Lopez-Cabrera M., Aroeira L.S. (2009). Chronic exposure of mouse peritoneum to peritoneal dialysis fluid: Structural and functional alterations of the peritoneal membrane. Perit. Dial. Int..

[112] Martin P., Gomez M., Lamana A., Cruz-Adalia A., Ramirez-Huesca M., Ursa M.A., Yanez-Mo M., Sanchez-Madrid F. (2010). CD69 Association with Jak3/Stat5 Proteins Regulates Th17 Cell Differentiation. Mol. Cell. Biol..

[113] Maksic D., Vasilijic S., Colic M., Stankovic-Popovic V., Bokonjic D. (2009). Systemic and intraperitoneal proinflammatory cytokine profiles in patients on continuous ambulatory peritoneal dialysis. Adv. Perit. Dial..

[114] Xiao J., Gong Y., Chen Y., Yu D., Wang X., Zhang X., Dou Y., Liu D., Cheng G., Lu S. (2017). IL-6 promotes epithelial-to-mesenchymal transition of human peritoneal mesothelial cells possibly through the JAK2/STAT3 signaling pathway. Am. J. Physiol. Renal Physiol..

[115] Pecoits-Filho R., Carvalho M.J., Stenvinkel P., Lindholm B., Heimbürger O. (2006). Systemic and intraperitoneal interleukin-6 system during the first year of peritoneal dialysis. Perit. Dial. Int..

[116] Schmitt C.P., Aufricht C. (2017). Is there such a thing as biocompatible peritoneal dialysis fluid?. Pediatr. Nephrol..

[117] Witowski J., Ksiązek K., Warnecke C., Kuźlan M., Korybalska K., Tayama H., Wiśniewska-Elnur J., Pawlaczyk K., Trómińska J., Brȩborowicz A. (2007). Role of mesothelial cell-derived granulocyte colony-stimulating factor in interleukin-17-induced neutrophil accumulation in the peritoneum. Kidney Int..

[118] Ruiz de Morales J.M.G., Puig L., Daudén E., Cañete J.D., Pablos J.L., Martín A.O., Juanatey C.G., Adán A., Montalbán X., Borruel N. (2020). Critical role of interleukin (IL)-17 in inflammatory and immune disorders: An updated review of the evidence focusing in controversies. Autoimmun. Rev..

[119] Yang X., Yang J., Xing X., Wan L., Li M. (2014). Increased frequency of Th17 cells in systemic sclerosis is related to disease activity and collagen overproduction. Arthritis Res. Ther..

[120] Liu M., Yang J., Xing X., Cui X., Li M. (2014). Interleukin-17A promotes functional activation of systemic sclerosis patient-derived dermal vascular smooth muscle cells by extracellular-regulated protein kinases signalling pathway. Arthritis Res. Ther..

[121] Witowski J., Pawlaczyk K., Breborowicz A., Scheuren A., Kuzlan-Pawlaczyk M., Wisniewska J., Polubinska A., Friess H., Gahl G.M., Frei U. (2000). IL-17 Stimulates Intraperitoneal Neutrophil Infiltration through the Release of GROα Chemokine from Mesothelial Cells. J. Immunol..

[122] Choi S.Y., Ryu H.M., Choi J.Y., Cho J.H., Kim C.D., Kim Y.L., Park S.H. (2017). The role of Toll-like receptor 4 in high-glucose-induced inflammatory and fibrosis markers in human peritoneal mesothelial cells. Int. Urol. Nephrol..

[123] Ruiz-Carpio V., Sandoval P., Aguilera A., Albar-Vizcaíno P., Perez-Lozano M.L., González-Mateo G.T., Acuña-Ruiz A., García-Cantalejo J., Botías P., Bajo M.A. (2017). Genomic reprograming analysis of the Mesothelial to mesenchymal transition identifies biomarkers in peritoneal dialysis patients. Sci. Rep..

[124] Yanez-Mo M., Lara-Pezzi E., Selgas R., Ramirez-Huesca M., Dominguez-Jimenez C., Jimenez-Heffernan J.A., Aguilera A., Sanchez-Tomero J.A., Bajo M.A., Alvarez V. (2003). Peritoneal dialysis and epithelial-to-mesenchymal transition of mesothelial cells. N. Engl. J. Med..

[125] Lopez-Cabrera M. (2014). Mesenchymal Conversion of Mesothelial Cells Is a Key Event in the Pathophysiology of the Peritoneum during Peritoneal Dialysis. Adv. Med..

[126] Del Peso G., Jiménez-Heffernan J.A., Bajo M.A., Aroeira L.S., Aguilera A., Fernández-Perpén A., Cirugeda A., Castro M.J., De Gracia R., Sánchez-Villanueva R. (2008). Epithelial-to-mesenchymal transition of mesothelial cells is an early event during peritoneal dialysis and is associated with high peritoneal transport. Kidney Int..

[127] Aroeira L.S., Loureiro J., Gonzalez-Mateo G.T., Fernandez-Millara V., del Peso G., Sanchez-Tomero J.A., Ruiz-Ortega M., Bajo M.A., Lopez-Cabrera M., Selgas R. (2008). Characterization of epithelial-to-mesenchymal transition of mesothelial cells in a mouse model of chronic peritoneal exposure to high glucose dialysate. Perit. Dial. Int..

[128] Strippoli R., Benedicto I., Lozano M.L.P., Cerezo A., López-Cabrera M., Del Pozo M.A. (2008). Epithelial-to-mesenchymal transition of peritoneal mesothelial cells is regulated by an ERK/NF-κB/Snail1 pathway. DMM Dis. Model. Mech..

[129] Wang Q., Yang X., Xu Y., Shen Z., Cheng H., Cheng F., Liu X., Wang R. (2018). RhoA/Rho-kinase triggers epithelial-mesenchymal transition in mesothelial cells and contributes to the pathogenesis of dialysis-related peritoneal fibrosis. Oncotarget.

[130] Liu Y., Dong Z., Liu H., Zhu J., Liu F., Chen G. (2015). Transition of mesothelial cell to fibroblast in peritoneal dialysis: EMT, stem cell or bystander?. Perit. Dial. Int..

[131] Aguilera A., Yáñez-Mo M., Selgas R., Sánchez-Madrid F., López-Cabrera M. (2005). Epithelial to mesenchymal transition as a triggering factor of peritoneal membrane fibrosis and angiogenesis in peritoneal dialysis patients. Curr. Opin. Investig. Drugs.

[132] Selgas R., Bajo A., Jimenez-Heffernan J.A., Sanchez-Tomero J.A., Del Peso G., Aguilera A., Lopez-Cabrera M. (2006). Epithelial-to-mesenchymal transition of the mesothelial cell--its role in the response of the peritoneum to dialysis. Nephrol. Dial. Transplant..

[133] Busnadiego O., Loureiro-Álvarez J., Sandoval P., Lagares D., Dotor J., Pérez-Lozano M.L., López-Armada M.J., Lamas S., López-Cabrera M., Rodríguez-Pascual F. (2015). A pathogenetic role for endothelin-1 in peritoneal dialysis-associated fibrosis. J. Am. Soc. Nephrol..

[134] Loureiro J., Schilte M., Aguilera A., Albar-Vizcaino P., Ramirez-Huesca M., Perez-Lozano M.L., Gonzalez-Mateo G., Aroeira L.S., Selgas R., Mendoza L. (2010). BMP-7 blocks mesenchymal conversion of mesothelial cells and prevents peritoneal damage induced by dialysis fluid exposure. Nephrol. Dial. Transplant..

[135] Chang J., Jiang Z., Zhang H., Zhu H., Zhou S.F., Yu X. (2011). NADPH oxidase-dependent formation of reactive oxygen species contributes to angiotensin II-induced epithelial-mesenchymal transition in rat peritoneal mesothelial cells. Int. J. Mol. Med..

[136] Hi B.L., Ha H. (2007). Mechanisms of epithelial-mesenchymal transition of peritoneal mesothelial cells during peritoneal dialysis. J. Korean Med. Sci..

[137] Xing X., Yang J., Yang X., Wei Y., Zhu L., Gao D., Li M. (2013). IL-17A induces endothelial inflammation in systemic sclerosis via the ERK signaling pathway. PLoS ONE.

[138] Karbach S., Croxford A.L., Oelze M., Schüler R., Minwegen D., Wegner J., Koukes L., Yogev N., Nikolaev A., Reißig S. (2014). Interleukin 17 drives vascular inflammation, endothelial dysfunction, and arterial hypertension in psoriasis-like skin disease. Arterioscler. Thromb. Vasc. Biol..

[139] Wu J., Saleh M.A., Kirabo A., Itani H.A., Montaniel K.R.C., Xiao L., Chen W., Mernaugh R.L., Cai H., Bernstein K.E. (2016). Immune activation caused by vascular oxidation promotes fibrosis and hypertension. J. Clin. Investig..

[140] Avila-Carrasco L., Majano P., Sánchez-Toméro J.A., Selgas R., López-Cabrera M., Aguilera A., González Mateo G. (2019). Natural Plants Compounds as Modulators of Epithelial-to-Mesenchymal Transition. Front. Pharmacol..

[141] Strippoli R., Benedicto I., Perez Lozano M.L., Pellinen T., Sandoval P., Lopez-Cabrera M., del Pozo M.A. (2012). Inhibition of transforming growth factor-activated kinase 1 (TAK1) blocks and reverses epithelial to mesenchymal transition of mesothelial cells. PLoS ONE.

[142] Strippoli R., Benedicto I., Foronda M., Perez-Lozano M.L., Sánchez-Perales S., López-Cabrera M., Del Pozo M.Á. (2010). p38 maintains E-cadherin expression by modulating TAK1-NF-κB during epithelial-to-mesenchymal transition. J. Cell Sci..

[143] Strippoli R., Loureiro J., Moreno V., Benedicto I., Pérez Lozano M.L., Barreiro O., Pellinen T., Minguet S., Foronda M., Osteso M.T. (2015). Caveolin-1 deficiency induces a MEK - ERK 1/2-Snail-1-dependent epithelial–mesenchymal transition and fibrosis during peritoneal dialysis. EMBO Mol. Med..

[144] Dudas P.L., Sague S.L., Elloso M.M., Farrell F.X. (2011). Proinflammatory/profibrotic effects of interleukin-17A on human proximal tubule epithelium. Nephron Exp. Nephrol..

[145] Gu K., Li M.-M., Shen J., Liu F., Cao J.-Y., Jin S., Yu Y. (2015). Interleukin-17-induced EMT promotes lung cancer cell migration and invasion via NF-κB/ZEB1 signal pathway. Am. J. Cancer Res..

[146] Orejudo M., García-Redondo A.B., Rodrigues-Diez R.R., Rodrigues-Díez R., Santos-Sanchez L., Tejera-Muñoz A., Egido J., Selgas R., Salaices M., Briones A.M. (2020). Interleukin-17A induces vascular remodeling of small arteries and blood pressure elevation. Clin. Sci. (Lond.).

[147] Wilson M.S., Madala S.K., Ramalingam T.R., Gochuico B.R., Rosas I.O., Cheever A.W., Wynn T.A. (2010). Bleomycin and IL-1β-mediated pulmonary fibrosis is IL-17A dependent. J. Exp. Med..

[148] Mi S., Li Z., Yang H.-Z., Liu H., Wang J.-P., Ma Y.-G., Wang X.-X., Liu H.-Z., Sun W., Hu Z.-W. (2011). Blocking IL-17A Promotes the Resolution of Pulmonary Inflammation and Fibrosis Via TGF-β1–Dependent and –Independent Mechanisms. J. Immunol..

[149] Cipolla E., Fisher A.J., Gu H., Mickler E.A., Agarwal M., Wilke C.A., Kim K.K., Moore B.B., Vittal R. (2017). IL-17A deficiency mitigates bleomycin-induced complement activation during lung fibrosis. FASEB J..

[150] Speeckaert R., Lambert J., Grine L., Van Gele M., De Schepper S., van Geel N. (2016). The many faces of interleukin-17 in inflammatory skin diseases. Br. J. Dermatol..

[151] Li J., Liu L., Zhao Q., Chen M. (2019). Role of Interleukin-17 in Pathogenesis of Intestinal Fibrosis in Mice. Dig. Dis. Sci..

[152] Mehrotra P., Collett J.A., McKinney S.D., Stevens J., Ivancic C.M., Basile D.P. (2017). IL-17 mediates neutrophil infiltration and renal fibrosis following recovery from ischemia reperfusion: Compensatory role of natural killer cells in athymic rats. Am. J. Physiol. Ren. Physiol..

[153] Ramani K., Tan R.J., Zhou D., Coleman B.M., Jawale C.V., Liu Y., Biswas P.S. (2018). IL-17 Receptor Signaling Negatively Regulates the Development of Tubulointerstitial Fibrosis in the Kidney. Mediat. Inflamm..

[154] Erbel C., Chen L., Bea F., Wangler S., Celik S., Lasitschka F., Wang Y., Böckler D., Katus H.A., Dengler T.J. (2009). Inhibition of IL-17A Attenuates Atherosclerotic Lesion Development in ApoE-Deficient Mice. J. Immunol..

[155] Truchetet M.E., Brembilla N.C., Montanari E., Lonati P., Raschi E., Zeni S., Fontao L., Meroni P.L., Chizzolini C. (2013). Interleukin-17A^+^ Cell Counts Are Increased in Systemic Sclerosis Skin and Their Number Is Inversely Correlated with the Extent of Skin Involvement. Arthritis Rheum..

[156] Nakashima T., Jinnin M., Yamane K., Honda N., Kajihara I., Makino T., Masuguchi S., Fukushima S., Okamoto Y., Hasegawa M. (2012). Impaired IL-17 Signaling Pathway Contributes to the Increased Collagen Expression in Scleroderma Fibroblasts. J. Immunol..

[157] Braun R.K., Ferrick C., Neubauer P., Sjoding M., Sterner-Kock A., Kock M., Putney L., Ferrick D.A., Hyde D.M., Love R.B. (2008). IL-17 producing γδ T cells are required for a controlled inflammatory response after bleomycin-induced lung injury. Inflammation.

[158] Ramani K., Garg A.V., Jawale C.V., Conti H.R., Whibley N., Jackson E.K., Shiva S.S., Horne W., Kolls J.K., Gaffen S.L. (2016). The Kallikrein-Kinin System: A Novel Mediator of IL-17-Driven Anti-Candida Immunity in the Kidney. PLoS Pathog..

[159] Loureiro J., Aguilera A., Selgas R., Sandoval P., Albar-Vizcaíno P., Pérez-Lozano M.L., Ruiz-Carpio V., Majano P.L., Lamas S., Rodríguez-Pascual F. (2011). Blocking TGF-β1 protects the peritoneal membrane from dialysate-induced damage. J. Am. Soc. Nephrol..

[160] Salzer W.L. (2018). Peritoneal dialysis-related peritonitis: Challenges and solutions. Int. J. Nephrol. Renovasc. Dis..

[161] Szeto C.C., Li P.K.T. (2019). Peritoneal dialysis–associated peritonitis. Clin. J. Am. Soc. Nephrol..

[162] Hurst S.M., Wilkinson T.S., McLoughlin R.M., Jones S., Horiuchi S., Yamamoto N., Rose-John S., Fuller G.M., Topley N., Jones S.A. (2001). IL-6 and its soluble receptor orchestrate a temporal switch in the pattern of leukocyte recruitment seen during acute inflammation. Immunity.

[163] Lai K.N., Lai K.B., Lam C.W.K., Chan T.M., Li F.K., Leung J.C.K. (2000). Changes of cytokine profiles during peritonitis in patients on continuous ambulatory peritoneal dialysis. Am. J. Kidney Dis..

[164] Chung D.R., Kasper D.L., Panzo R.J., Chtinis T., Grusby M.J., Sayegh M.H., Tzianabos A.O. (2003). CD4^+^ T Cells Mediate Abscess Formation in Intra-abdominal Sepsis by an IL-17-Dependent Mechanism. J. Immunol..

[165] Murphy A.G., O’Keeffe K.M., Lalor S.J., Maher B.M., Mills K.H.G., McLoughlin R.M. (2014). Staphylococcus aureus Infection of Mice Expands a Population of Memory γδ T Cells That Are Protective against Subsequent Infection. J. Immunol..

[166] Li J., Zhang Y., Lou J., Zhu J., He M., Deng X., Cai Z. (2012). Neutralisation of Peritoneal IL-17A Markedly Improves the Prognosis of Severe Septic Mice by Decreasing Neutrophil Infiltration and Proinflammatory Cytokines. PLoS ONE.

[167] Wang H.H., Lin C.Y. (2005). Interleukin-12 and -18 levels in peritoneal dialysate effluent correlate with the outcome of peritonitis in patients undergoing peritoneal dialysis: Implications for the type I/type II T-cell immune response. Am. J. Kidney Dis..

[168] Lin C.Y., Roberts G.W., Kift-Morgan A., Donovan K.L., Topley N., Eberl M. (2013). Pathogen-specific local immune fingerprints diagnose bacterial infection in peritoneal dialysis patients. J. Am. Soc. Nephrol..

[169] Zhang J., Friberg I.M., Kift-Morgan A., Parekh G., Morgan M.P., Liuzzi A.R., Lin C.Y., Donovan K.L., Colmont C.S., Morgan P.H. (2017). Machine-learning algorithms define pathogen-specific local immune fingerprints in peritoneal dialysis patients with bacterial infections. Kidney Int..

[170] Habib S.M., Abrahams A.C., Korte M.R., Zietse R., De Vogel L.L., Boer W.H., Dendooven A., Van Groningen M.C.C., Betjes M.G.H. (2015). CD4-positive T cells and M2 macrophages dominate the peritoneal infiltrate of patients with encapsulating peritoneal sclerosis. PLoS ONE.

[171] Hu W., Jiang Z., Zhang Y., Liu Q., Fan J., Luo N., Dong X., Yu X. (2012). Characterization of infiltrating macrophages in high glucose-induced peritoneal fibrosis in rats. Mol. Med. Rep..

[172] Fernandez de Castro M., Selgas R., Jimenez C., Auxiliadora Bajo M., Martinez V., Romero J.R., de Alvaro F., Vara F. (1994). Cell populations present in the nocturnal peritoneal effluent of patients on continuous ambulatory peritoneal dialysis and their relationship with peritoneal function and incidence of peritonitis. Perit. Dial. Int..

[173] Anders H.J., Ryu M. (2011). Renal microenvironments and macrophage phenotypes determine progression or resolution of renal inflammation and fibrosis. Kidney Int..

[174] Meng X.M., Mak T.S.K., Lan H.Y. (2019). Macrophages in Renal Fibrosis. Advances in Experimental Medicine and Biology.

[175] Mantovani A., Sica A. (2010). Macrophages, innate immunity and cancer: Balance, tolerance, and diversity. Curr. Opin. Immunol..

[176] Mantovani A., Sozzani S., Locati M., Allavena P., Sica A. (2002). Macrophage polarization: Tumor-associated macrophages as a paradigm for polarized M2 mononuclear phagocytes. Trends Immunol..

[177] Wang Y., Harris D.C.H. (2011). Macrophages in renal disease. J. Am. Soc. Nephrol..

[178] Kinsey G.R. (2014). Macrophage dynamics in AKI to CKD progression. J. Am. Soc. Nephrol..

[179] Mosser D.M., Edwards J.P. (2008). Exploring the full spectrum of macrophage activation. Nat. Rev. Immunol..

[180] Liao C.-T., Andrews R., Wallace L.E., Khan M.W.A., Kift-Morgan A., Topley N., Fraser D.J., Taylor P.R. (2017). Peritoneal macrophage heterogeneity is associated with different peritoneal dialysis outcomes. Kidney Int..

[181] Fieren M.W.J.A. (1996). Mechanisms Regulating Cytokine Release from Peritoneal Macrophages during Continuous Ambulatory Peritoneal Dialysis. Blood Purif..

[182] Bellon T., Martinez V., Lucendo B., del Peso G., Castro M.J., Aroeira L.S., Rodriguez-Sanz A., Ossorio M., Sanchez-Villanueva R., Selgas R. (2011). Alternative activation of macrophages in human peritoneum: Implications for peritoneal fibrosis. Nephrol. Dial. Transplant.

[183] Ossorio M., Martinez V., Bajo M.-A., Del Peso G., Castro M.-J., Romero S., Selgas R., Bellon T. (2018). Prominent Levels of the Profibrotic Chemokine CCL18 during Peritonitis: In Vitro Downregulation by Vitamin D Receptor Agonists. Biomed Res. Int..

[184] Schutyser E. (2005). Involvement of CC chemokine ligand 18 (CCL18) in normal and pathological processes. J. Leukoc. Biol..

[185] Chen Y.T., Hsu H., Lin C.C., Pan S.Y., Liu S.Y., Wu C.F., Tsai P.Z., Liao C.T., Cheng H.T., Chiang W.C. (2020). Inflammatory macrophages switch to CCL17-expressing phenotype and promote peritoneal fibrosis. J. Pathol..

[186] Li Q., Zheng M., Liu Y., Sun W., Shi J., Ni J., Wang Q. (2018). A pathogenetic role for M1 macrophages in peritoneal dialysis-associated fibrosis. Mol. Immunol..

[187] Wang J., Jiang Z.P., Su N., Fan J.J., Ruan Y.P., Peng W.X., Li Y.F., Yu X.Q. (2013). The role of peritoneal alternatively activated macrophages in the process of peritoneal fibrosis related to peritoneal dialysis. Int. J. Mol. Sci..

[188] Sikorska- Wiśniewska M., Mika A., Śledziński T., Małgorzewicz S., Stepnowski P., Rutkowski B., Chmielewski M. (2017). Disorders of serum omega-3 fatty acid composition in dialyzed patients, and their associations with fat mass. Ren. Fail..

[189] Tang H., Zhu X., Gong C., Liu H., Liu F. (2019). Protective effects and mechanisms of omega-3 polyunsaturated fatty acid on intestinal injury and macrophage polarization in peritoneal dialysis rats. Nephrology.

[190] Sergejeva S., Linden A. (2009). Impact of IL-17 on Cells of the Monocyte Lineage in Health and Disease. Endocr. Metab. Immune Disord. Drug Targets.

[191] Shahrara S., Pickens S.R., Dorfleutner A., Pope R.M. (2009). IL-17 Induces Monocyte Migration in Rheumatoid Arthritis. J. Immunol..

[192] Shen J., Sun X., Pan B., Cao S., Cao J., Che D., Liu F., Zhang S., Yu Y. (2018). IL-17 induces macrophages to M2-like phenotype via NF-κB. Cancer Manag. Res..

[193] Liu L., Ge D., Ma L., Mei J., Liu S., Zhang Q., Ren F., Liao H., Pu Q., Wang T. (2012). Interleukin-17 and prostaglandin E2 are involved in formation of an M2 macrophage-dominant microenvironment in lung cancer. J. Thorac. Oncol..

[194] Shi S., Zhang Q., Atsuta I., Liu S., Chen C., Shi S., Le A.D. (2013). IL-17-mediated M1/M2 macrophage alteration contributes to pathogenesis of bisphosphonate-related osteonecrosis of the jaws. Clin. Cancer Res..

[195] Miller J.E., Ahn S.H., Marks R.M., Monsanto S.P., Fazleabas A.T., Koti M., Tayade C. (2020). IL-17A Modulates Peritoneal Macrophage Recruitment and M2 Polarization in Endometriosis. Front. Immunol..

[196] Nishikawa K., Seo N., Torii M., Ma N., Muraoka D., Tawara I., Masuya M., Tanaka K., Takei Y., Shiku H. (2014). Interleukin-17 induces an atypical M2-Like macrophage subpopulation that regulates intestinal inflammation. PLoS ONE.

[197] Tian L.X., Tang X., Zhu J.Y., Zhang W., Tang W.Q., Yan J., Xu X., Liang H.P. (2020). Cytochrome P450 1A1 enhances Arginase-1 expression, which reduces LPS-induced mouse peritonitis by targeting JAK1/STAT6. Cell. Immunol..

[198] Stengel S., Quickert S., Lutz P., Ibidapo-Obe O., Steube A., Köse-Vogel N., Yarbakht M., Reuken P.A., Busch M., Brandt A. (2020). Peritoneal Level of CD206 Associates With Mortality and an Inflammatory Macrophage Phenotype in Patients With Decompensated Cirrhosis and Spontaneous Bacterial Peritonitis. Gastroenterology.

[199] Aroeira L.S., Aguilera A., Selgas R., Ramírez-Huesca M., Pérez-Lozano M.L., Cirugeda A., Bajo M.A., Del Peso G., Sánchez-Tomero J.A., Jiménez-Heffernan J.A. (2005). Mesenchymal conversion of mesothelial cells as a mechanism responsible for high solute transport rate in peritoneal dialysis: Role of vascular endothelial growth factor. Am. J. Kidney Dis..

[200] Williams J.D., Craig K.J., Topley N., Von Ruhland C., Fallon M., Newman G.R., Mackenzie R.K., Williams G.T. (2002). Morphologic changes in the peritoneal membrane of patients with renal disease. J. Am. Soc. Nephrol..

[201] Numata M., Nakayama M., Nimura S., Kawakami M., Lindholm B., Kawaguchi Y. (2003). Association between an increased surface area of peritoneal microvessels and a high peritoneal solute transport rate. Perit. Dial. Int..

[202] Pérez-Lozano M.L., Sandoval P., Rynne-Vidal Á., Aguilera A., Jiménez-Heffernan J.A., Albar-Vizcaíno P., Majano P.L., Sánchez-Tomero J.A., Selgas R., López-Cabrera M. (2013). Functional Relevance of the Switch of VEGF Receptors/Co-Receptors during Peritoneal Dialysis-Induced Mesothelial to Mesenchymal Transition. PLoS ONE.

[203] Numasaki M., Fukushi J.I., Ono M., Narula S.K., Zavodny P.J., Kudo T., Robbins P.D., Tahara H., Lotze M.T. (2003). Interleukin-17 promotes angiogenesis and tumor growth. Blood.

[204] Wakita D., Sumida K., Iwakura Y., Nishikawa H., Ohkuri T., Chamoto K., Kitamura H., Nishimura T. (2010). Tumor-infiltrating IL-17-producing γδ T cells support the progression of tumor by promoting angiogenesis. Eur. J. Immunol..

[205] Liu J., Duan Y., Cheng X., Chen X., Xie W., Long H., Lin Z., Zhu B. (2011). IL-17 is associated with poor prognosis and promotes angiogenesis via stimulating VEGF production of cancer cells in colorectal carcinoma. Biochem. Biophys. Res. Commun..

[206] Huang Q., Duan L., Qian X., Fan J., Lv Z., Zhang X., Han J., Wu F., Guo M., Hu G. (2016). IL-17 Promotes Angiogenic Factors IL-6, IL-8, and Vegf Production via Stat1 in Lung Adenocarcinoma. Sci. Rep..

[207] Pan B., Shen J., Cao J., Zhou Y., Shang L., Jin S., Cao S., Che D., Liu F., Yu Y. (2015). Interleukin-17 promotes angiogenesis by stimulating VEGF production of cancer cells via the STAT3/GIV signaling pathway in non-small-cell lung cancer. Sci. Rep..

[208] Hu B., Wang J., Wu X., Chen Y., Yuan W., Chen H. (2017). Interleukin-17 upregulates vascular endothelial growth factor by activating the JAK/STAT pathway in nucleus pulposus cells. Jt. Bone Spine.

[209] Keeley E.C., Mehrad B., Strieter R.M. (2011). Chemokines as mediators of tumor angiogenesis and neovascularization. Exp. Cell Res..

[210] Addison C.L., Daniel T.O., Burdick M.D., Liu H., Ehlert J.E., Xue Y.Y., Buechi L., Walz A., Richmond A., Strieter R.M. (2000). The CXC Chemokine Receptor 2, CXCR2, Is the Putative Receptor for ELR + CXC Chemokine-Induced Angiogenic Activity. J. Immunol..

[211] Liu L., Sun H., Wu S., Tan H., Sun Y., Liu X., Si S., Xu L., Huang J., Zhou W. (2019). IL-17A promotes CXCR2-dependent angiogenesis in a mouse model of liver cancer. Mol. Med. Rep..

[212] Wei Z.W., Xia G.K., Wu Y., Chen W., Xiang Z., Schwarz R.E., Brekken R.A., Awasthi N., He Y.L., Zhang C.H. (2015). CXCL1 promotes tumor growth through VEGF pathway activation and is associated with inferior survival in gastric cancer. Cancer Lett..

[213] Martin D., Galisteo R., Gutkind J.S. (2008). CXCL8/IL8 stimulates VEGF expression and the autocrine activation of VEGFR2 in endothelial cells by activating NFkappa B through the CBM (Carma3/Bcl10/Matl1) complex. J. Biol. Chem..

[214] Ichiyama K., Yoshida H., Wakabayashi Y., Chinen T., Saeki K., Nakaya M., Takaesu G., Hori S., Yoshimura A., Kobayashi T. (2008). Foxp3 inhibits RORγt-mediated IL-17A mRNA transcription through direct interaction with RORγt. J. Biol. Chem..

[215] Kim J.M., Rasmussen J.P., Rudensky A.Y. (2007). Regulatory T cells prevent catastrophic autoimmunity throughout the lifespan of mice. Nat. Immunol..

[216] Kondělková K., Vokurková D., Krejsek J., Borská L., Fiala Z., Ctirad A. (2010). Regulatory T cells (TREG) and their roles in immune system with respect to immunopathological disorders. Acta Med. (Hradec Kralove).

[217] Noack M., Miossec P. (2014). Th17 and regulatory T cell balance in autoimmune and inflammatory diseases. Autoimmun. Rev..

[218] Zhou L., Lopes J.E., Chong M.M.W., Ivanov I.I., Min R., Victora G.D., Shen Y., Du J., Rubtsov Y.P., Rudensky A.Y. (2008). TGF-Β-induced Foxp3 inhibits TH17 cell differentiation by antagonizing RORγt function. Nature.

[219] Cravedi P., Remuzzi G., Ruggenenti P. (2011). Targeting the Renin Angiotensin System in Dialysis Patients. Semin. Dial..

[220] Noh H., Ha H., Yu M.R., Kim Y.O., Kim J.H., Lee H.B. (2005). Angiotensin II mediates high glucose-induced TGF-beta1 and fibronectin upregulation in HPMC through reactive oxygen species. Perit. Dial. Int..

[221] Nessim S.J., Perl J., Bargman J.M. (2010). The renin-angiotensin-aldosterone system in peritoneal dialysis: Is what is good for the kidney also good for the peritoneum. Kidney Int..

[222] Nakamoto H., Imai H., Fukushima R., Ishida Y., Yamanouchi Y., Suzuki H. (2008). Role of the renin-angiotensin system in the pathogenesis of peritoneal fibrosis. Perit. Dial. Int..

[223] Kyuden Y., Ito T., Masaki T., Yorioka N., Kohno N. (2005). Tgf-beta1 induced by high glucose is controlled by angiotensin-converting enzyme inhibitor and angiotensin II receptor blocker on cultured human peritoneal mesothelial cells. Perit. Dial. Int..

[224] Duman S., Gunal A.I., Sen S., Asci G., Ozkahya M., Terzioglu E., Akcicek F., Atabay G. (2001). Does enalapril prevent peritoneal fibrosis induced by hypertonic (3.86%) peritoneal dialysis solution?. Perit. Dial. Int..

[225] Duman S., Sen S., Duman C., Oreopoulos D.G. (2005). Effect of valsartan versus lisinopril on peritoneal sclerosis in rats. Int. J. Artif. Organs.

[226] Kolesnyk I., Dekker F.W., Noordzij M., le Cessie S., Struijk D.G., Krediet R.T. (2007). Impact of ACE inhibitors and AII receptor blockers on peritoneal membrane transport characteristics in long-term peritoneal dialysis patients. Perit. Dial. Int..

[227] Zhang L., Zeng X., Fu P., Wu H.M. (2014). Angiotensin-converting enzyme inhibitors and angiotensin receptor blockers for preserving residual kidney function in peritoneal dialysis patients. Cochrane Database Syst. Rev..

[228] Phatthanasobhon S., Nochaiwong S., Thavorn K., Noppakun K., Panyathong S., Suteeka Y., Hutton B., Sood M.M., Knoll G.A., Ruengorn C. (2019). Effectiveness of Renin-Angiotensin-Aldosterone System Blockade on Residual Kidney Function and Peritoneal Membrane Function in Peritoneal Dialysis Patients: A Network Meta-Analysis. Sci. Rep..

[229] Platten M., Youssef S., Eun M.H., Ho P.P., Han M.H., Lanz T.V., Phillips L.K., Goldstein M.J., Bhat R., Raine C.S. (2009). Blocking angiotensin-converting enzyme induces potent regulatory T cells and modulates TH1- and TH17-mediated autoimmunity. Proc. Natl. Acad. Sci. USA.

[230] Uzawa A., Mori M., Taniguchi J., Kuwabara S. (2014). Modulation of the kallikrein/kinin system by the angiotensin-converting enzyme inhibitor alleviates experimental autoimmune encephalomyelitis. Clin. Exp. Immunol..

[231] Weber J., Tiriveedhi V., Takenaka M., Lu W., Hachem R., Trulock E., Patterson G.A., Mohanakumar T. (2012). Inhibition of renin angiotensin aldosterone system causes abrogation of obliterative airways disease through inhibition of tumor necrosis factor-αdependant interleukin-17. J. Heart Lung Transplant..

[232] Suda N., Moriyama K., Ganburgedc G. (2013). Effect of angiotensin II receptor blocker on experimental periodontitis in a mouse model of marfan syndrome. Infect. Immun..

[233] Coelho dos Santos J.S., Menezes C.A.S., Villani F.N.A., Magalhães L.M.D., Scharfstein J., Gollob K.J., Dutra W.O. (2010). Captopril increases the intensity of monocyte infection by Trypanosoma cruzi and induces human T helper type 17 cells. Clin. Exp. Immunol..

[234] Oesterle A., Laufs U., Liao J.K. (2017). Pleiotropic Effects of Statins on the Cardiovascular System. Circ. Res..

[235] Wang C.Y., Liu P.Y., Liao J.K. (2008). Pleiotropic effects of statin therapy: Molecular mechanisms and clinical results. Trends Mol. Med..

[236] Navaneethan S.D., Nigwekar S.U., Perkovic V., Johnson D.W., Craig J.C., Strippoli G.F. (2013). HMG CoA reductase inhibitors (statins) for dialysis patients. Cochrane Database Syst. Rev..

[237] Obialo C.I., Ofili E.O., Norris K.C. (2018). Statins and cardiovascular disease outcomes in chronic kidney disease: Reaffirmation vs. repudiation. Int. J. Environ. Res. Public Health.

[238] Chen Z., Qureshi A.R., Parini P., Hurt-Camejo E., Ripsweden J., Brismar T.B., Barany P., Jaminon A.M., Schurgers L.J., Heimbürger O. (2017). Does statins promote vascular calcification in chronic kidney disease?. Eur. J. Clin. Investig..

[239] Kumar S., Raftery M., Yaqoob M., Fan S.L.-S. (2007). Anti-inflammatory effects of 3-hydroxy-3-methylglutaryl coenzyme a reductase inhibitors (statins) in peritoneal dialysis patients. Perit. Dial. Int..

[240] Carrión B., Pérez-Martínez F.C., Monteagudo S., Pérez-Carrión M.D., Gómez-Roldán C., Ceña V., Pérez-Martínez J. (2011). Atorvastatin reduces high glucose toxicity in rat peritoneal mesothelial cells. Perit. Dial. Int..

[241] Chang T.I., Kang H.Y., Kim K.S., Lee S.H., Nam B.Y., Paeng J., Kim S., Park J.T., Yoo T.H., Kang S.W. (2014). The effect of statin on epithelial-mesenchymal transition in peritoneal mesothelial cells. PLoS ONE.

[242] Zhang L., Liu J., Liu Y., Xu Y., Zhao X., Qian J., Sun B., Xing C. (2015). Fluvastatin inhibits the expression of fibronectin in human peritoneal mesothelial cells induced by high-glucose peritoneal dialysis solution via SGK1 pathway. Clin. Exp. Nephrol..

[243] Duman S., Sen S., Sözmen E.Y., Oreopoulos D.G. (2005). Atorvastatin improves peritoneal sclerosis induced by hypertonic PD solution in rats. Int. J. Artif. Organs.

[244] Zhang X., Markovic-Plese S. (2008). Statins’ immunomodulatory potential against Th17 cell-mediated autoimmune response. Immunol. Res..

[245] Zhang X., Jin J., Peng X., Ramgolam V.S., Markovic-Plese S. (2008). Simvastatin Inhibits IL-17 Secretion by Targeting Multiple IL-17-Regulatory Cytokines and by Inhibiting the Expression of IL-17 Transcription Factor RORC in CD4^+^ Lymphocytes. J. Immunol..

[246] Frostegård J., Zhang Y., Sun J., Yan K., Liu A. (2016). Oxidized Low-Density Lipoprotein (OxLDL)-Treated Dendritic Cells Promote Activation of T Cells in Human Atherosclerotic Plaque and Blood, Which Is Repressed by Statins: MicroRNA let-7c Is Integral to the Effect. J. Am. Heart Assoc..

[247] Li Z., Chen L., Niu X., Liu J., Ping M., Li R., Xie X., Guo L. (2012). Immunomodulatory synergy by combining atorvastatin and rapamycin in the treatment of experimental autoimmune encephalomyelitis (EAE). J. Neuroimmunol..

[248] Aktunc E., Kayhan B., Arasli M., Gun B.D., Barut F. (2011). The effect of atorvastatin and its role on systemic cytokine network in treatment of acute experimental colitis. Immunopharmacol. Immunotoxicol..

[249] Han S.H., Kang E.W., Yoon S.-J., Yoon H.S., Lee H.C., Yoo T.H., Choi K.H., Han D.-S., Kang S.-W. (2011). Combined vascular effects of HMG-CoA reductase inhibitor and angiotensin receptor blocker in non-diabetic patients undergoing peritoneal dialysis. Nephrol. Dial. Transpl..

[250] Liu Z., Zhao Y., Wei F., Ye L., Lu F., Zhang H., Diao Y., Song H., Qi Z. (2014). Treatment with telmisartan/rosuvastatin combination has a beneficial synergistic effect on ameliorating Th17/Treg functional imbalance in hypertensive patients with carotid atherosclerosis. Atherosclerosis.

[251] Ma X., Liu S., Li T., Yuan H. (2019). Intensive statin treatment ameliorate the Th17/Treg functional imbalance in patients with non-ST elevation acute coronary syndrome underwent percutaneous coronary intervention. Clin. Cardiol..

[252] Rostamzadeh D., Yousefi M., Haghshenas M.R., Ahmadi M., Dolati S., Babaloo Z. (2019). mTOR Signaling pathway as a master regulator of memory CD8^+^ T-cells, Th17, and NK cells development and their functional properties. J. Cell. Physiol..

[253] Ikejiri A., Nagai S., Goda N., Kurebayashi Y., Osada-Oka M., Takubo K., Suda T., Koyasu S. (2012). Dynamic regulation of Th17 differentiation by oxygen concentrations. Int. Immunol..

[254] Hou H., Miao J., Cao R., Han M., Sun Y., Liu X., Guo L. (2017). Rapamycin Ameliorates Experimental Autoimmune Encephalomyelitis by Suppressing the mTOR-STAT3 Pathway. Neurochem. Res..

[255] Hou H., Cao R., Quan M., Sun Y., Sun H., Zhang J., Li B., Guo L., Song X. (2018). Rapamycin and fingolimod modulate Treg/Th17 cells in experimental autoimmune encephalomyelitis by regulating the Akt-mTOR and MAPK/ERK pathways. J. Neuroimmunol..

[256] Patel P., Sekiguchi Y., Oh K.H., Patterson S.E., Kolb M.R.J., Margetts P.J. (2010). Smad3-dependent and -independent pathways are involved in peritoneal membrane injury. Kidney Int..

[257] Sekiguchi Y., Zhang J., Patterson S., Liu L., Hamada C., Tomino Y., Margetts P.J. (2012). Rapamycin inhibits transforming growth factor β-induced peritoneal angiogenesis by blocking the secondary hypoxic response. J. Cell. Mol. Med..

[258] Aguilera A., Aroeira L.S., Ramírez-Huesca M., Pérez-Lozano M.L., Cirugeda A., Bajo M.A., Del Peso G., Valenzuela-Fernández A., Sánchez-Tomero J.A., López-Cabrera M. (2005). Effects of rapamycin on the epithelial-to-mesenchymal transition of human peritoneal mesothelial cells. Int. J. Artif. Organs.

[259] Gonzalez-Mateo G.T., Aguirre A.R., Loureiro J., Abensur H., Sandoval P., Sanchez-Tomero J.A., del Peso G., Jimenez-Heffernan J.A., Ruiz-Carpio V., Selgas R. (2015). Rapamycin Protects from Type-I Peritoneal Membrane Failure Inhibiting the Angiogenesis, Lymphangiogenesis, and Endo-MT. Biomed Res. Int..

[260] Xiang S., Li M., Xie X., Xie Z., Zhou Q., Tian Y., Lin W., Zhang X., Jiang H., Shou Z. (2016). Rapamycin inhibits epithelial-to-mesenchymal transition of peritoneal mesothelium cells through regulation of Rho GTPases. FEBS J..

[261] Xu T., Xie J.Y., Wang W.M., Ren H., Chen N. (2012). Impact of rapamycin on peritoneal fibrosis and transport function. Blood Purif..

[262] Liu J., Jiang C.-M., Feng Y., Zhu W., Jin B., Xia Y.-Y., Zhang Q.-Y., Xu P.-F., Zhang M. (2019). Rapamycin inhibits peritoneal fibrosis by modifying lipid homeostasis in the peritoneum. Am. J. Transl. Res..

[263] Aroeira L.S., Lara-Pezzi E., Loureiro J., Aguilera A., Ramírez-Huesca M., González-Mateo G., Pérez-Lozano M.L., Albar-Vizcaíno P., Bajo M.A., Del Peso G. (2009). Cyclooxygenase-2 mediates dialysate-Lnduced alterations of the peritoneal membrane. J. Am. Soc. Nephrol..

[264] Fabbrini P., Schilte M.N., Zareie M., ter Wee P.M., Keuning E.D., Beelen R.H.J., van den Born J. (2009). Celecoxib treatment reduces peritoneal fibrosis and angiogenesis and prevents ultrafiltration failure in experimental peritoneal dialysis. Nephrol. Dial. Transplant..

[265] Kim S.B., Kim S.H., Chang J.W., Lee S.K., Min W.K., Chi H.S., Park J.S. (2004). Effects of celecoxib on high-sensitivity C-reactive protein in chronic peritoneal dialysis patients. Ren. Fail..

[266] Li H., Alyce Bradbury J., Dackor R.T., Edin M.L., Graves J.P., DeGraff L.M., Wang P.M., Bortner C.D., Maruoka S., Lih F.B. (2011). Cyclooxygenase-2 regulates Th17 cell differentiation during allergic lung inflammation. Am. J. Respir. Crit. Care Med..

[267] Paulissen S.M.J., van Hamburg J.P., Davelaar N., Asmawidjaja P.S., Hazes J.M.W., Lubberts E. (2013). Synovial Fibroblasts Directly Induce Th17 Pathogenicity via the Cyclooxygenase/Prostaglandin E_2_ Pathway, Independent of IL-23. J. Immunol..

[268] Perazella M.A. (2002). COX-2 selective inhibitors: Analysis of the renal effects. Expert Opin. Drug Saf..

[269] Lotz L., Burgdorf S., Dani I., Saijo K., Flossdorf J., Hucke S., Alferink J., Novak N., Beyer M., Mayer G. (2009). The nuclear receptor PPARγ selectively inhibits Th17 differentiation in a T cell-intrinsic fashion and suppresses CNS autoimmunity. J. Exp. Med..

[270] Park S.J., Lee K.S., Kim S.R., Min K.H., Choe Y.H., Moon H., Chae H.J., Yoo W.H., Lee Y.C. (2009). Peroxisome Proliferator-Activated Receptor γ Agonist Down-Regulates IL-17 Expression in a Murine Model of Allergic Airway Inflammation. J. Immunol..

[271] Zhao Y., Huang Y., He J., Li C., Deng W., Ran X., Wang D. (2014). Rosiglitazone, a peroxisome proliferator-activated receptor-γ agonist, attenuates airway inflammation by inhibiting the proliferation of effector T cells in a murine model of neutrophilic asthma. Immunol. Lett..

[272] Farnesi-de-Assunção T.S., Alves C.F., Carregaro V., de Oliveira J.R., da Silva C.A.T., Cheraim A.B., Cunha F.Q., Napimoga M.H. (2012). PPAR-γ agonists, mainly 15d-PGJ 2, reduce eosinophil recruitment following allergen challenge. Cell. Immunol..

[273] Sandoval P., Loureiro J., González-Mateo G., Pérez-Lozano M.L., Maldonado-Rodríguez A., Sánchez-Tomero J.A., Mendoza L., Santamaría B., Ortiz A., Ruíz-Ortega M. (2010). PPAR-γ agonist rosiglitazone protects peritoneal membrane from dialysis fluid-induced damage. Lab. Investig..

[274] Zhang Y.F., Wang Q., Su Y.Y., Wang J.L., Hua B.J., Yang S., Feng J.X., Li H.Y. (2017). PPAR-γ agonist rosiglitazone protects rat peritoneal mesothelial cells against peritoneal dialysis solution-induced damage. Mol. Med. Rep..

[275] Zhang Y., Feng J., Wang Q., Zhao S., Xu J., Li H. (2018). PPAR-γ agonist rosiglitazone ameliorates peritoneal deterioration in peritoneal dialysis rats with LPS-induced peritonitis through up-regulation of AQP-1 and ZO-1. Biosci. Rep..

[276] Pioglitazone Actavis | European Medicines Agency. https://www.ema.europa.eu/en/medicines/human/EPAR/pioglitazone-actavis.

[277] Aufricht C., Endemann M., Bidmon B., Arbeiter K., Mueller T., Regele H., Herkner K., Eickelberg O. (2001). Peritoneal dialysis fluids induce the stress response in human mesothelial cells. Perit. Dial. Int..

[278] Kratochwill K., Boehm M., Herzog R., Lichtenauer A.M., Salzer E., Lechner M., Kuster L., Bergmeister K., Rizzi A., Mayer B. (2012). Alanyl-glutamine dipeptide restores the cytoprotective stress proteome of mesothelial cells exposed to peritoneal dialysis fluids. Nephrol. Dial. Transplant..

[279] Newsholme P. (2001). Why is L-glutamine metabolism important to cells of the immune system in health, postinjury, surgery or infection?. J. Nutr..

[280] Bender T.O., Böhm M., Kratochwill K., Lederhuber H., Endemann M., Bidmon B., Aufricht C. (2010). HSP-mediated cytoprotection of mesothelial cells in experimental acute peritoneal dialysis. Perit. Dial. Int..

[281] Kratochwill K., Boehm M., Herzog R., Gruber K., Lichtenauer A.M., Kuster L., Csaicsich D., Gleiss A., Alper S.L., Aufricht C. (2016). Addition of alanyl-glutamine to dialysis fluid restores peritoneal cellular stress responses ± a first-in-man trial. PLoS ONE.

[282] Herzog R., Kuster L., Becker J., Gluexam T., Pils D., Spittler A., Bhasin M.K., Alper S.L., Vychytil A., Aufricht C. (2017). Functional and Transcriptomic Characterization of Peritoneal Immune-Modulation by Addition of Alanyl-Glutamine to Dialysis Fluid. Sci. Rep..

[283] Ferrantelli E., Liappas G., Vila Cuenca M., Keuning E.D., Foster T.L., Vervloet M.G., Lopéz-Cabrera M., Beelen R.H.J. (2016). The dipeptide alanyl-glutamine ameliorates peritoneal fibrosis and attenuates IL-17 dependent pathways during peritoneal dialysis. Kidney Int..

[284] González-Mateo G.T., Fernández-Míllara V., Bellón T., Liappas G., Ruiz-Ortega M., López-Cabrera M., Selgas R., Aroeira L.S. (2014). Paricalcitol reduces peritoneal fibrosis in mice through the activation of regulatory T cells and reduction in IL-17 production. PLoS ONE.

[285] Stavenuiter A.W.D., Farhat K., Vila Cuenca M., Schilte M.N., Keuning E.D., Paauw N.J., ter Wee P.M., Beelen R.H.J., Vervloet M.G. (2015). Protective Effects of Paricalcitol on Peritoneal Remodeling during Peritoneal Dialysis. Biomed Res. Int..

[286] Kang S.H., Kim S.O., Cho K.H., Park J.W., Yoon K.W., Do J.Y. (2014). Paricalcitol ameliorates epithelial-to-mesenchymal transition in the peritoneal mesothelium. Nephron Exp. Nephrol..

[287] Ko J., Kang H.J., Kim D.A., Ryu E.S., Yu M., Lee H., Lee H.K., Ryu H.M., Park S.H., Kim Y.L. (2019). Paricalcitol attenuates TGF-b1-induced phenotype transition of human peritoneal mesothelial cells (HPMCs) via modulation of oxidative stress and NLRP3 inflammasome. FASEB J..

[288] Jerónimo T., Malho Guedes A., Del Peso G., Silva A.P., Selgas R., Bajo M.A., Neves P.L. (2018). Paricalcitol and Peritoneal Protein Loss in Peritoneal Dialysis: A Double-Center Study. Blood Purif..

[289] Morgado-Pascual J.L., Marchant V., Rodrigues-Diez R., Dolade N., Suarez-Alvarez B., Kerr B., Valdivielso J.M., Ruiz-Ortega M., Rayego-Mateos S. (2018). Epigenetic modification mechanisms involved in inflammation and fibrosis in renal pathology. Mediat. Inflamm..

[290] Gebert L.F.R., MacRae I.J. (2019). Regulation of microRNA function in animals. Nat. Rev. Mol. Cell Biol..

[291] Yanai K., Ishii H., Aomatsu A., Ishibashi K., Morishita Y. (2018). MicroRNAs in peritoneal fibrosis: A systematic review. Discov. Med..

[292] Chen J., Kam-Tao P., Kwan B.C.H., Chow K.M., Lai K.B., Luk C.C.W., Szeto C.C. (2012). Relation between microRNA expression in peritoneal dialysis effluent and peritoneal transport characteristics. Dis. Markers.

[293] Khan D., Ahmed S.A. (2015). Regulation of IL-17 in autoimmune diseases by transcriptional factors and microRNAs. Front. Genet..

[294] Mai J., Virtue A., Maley E., Tran T., Yin Y., Meng S., Pansuria M., Jiang X., Wang H., Yang X.F. (2012). MicroRNAs and other mechanisms regulate interleukin-17 cytokines and receptors. Front. Biosci. (Elite Ed.).

[295] Murugaiyan G., Da Cunha A.P., Ajay A.K., Joller N., Garo L.P., Kumaradevan S., Yosef N., Vaidya V.S., Weiner H.L. (2015). MicroRNA-21 promotes Th17 differentiation and mediates experimental autoimmune encephalomyelitis. J. Clin. Investig..

[296] Yao S.X., Zhang G.S., Cao H.X., Song G., Li Z.T., Zhang W.T. (2015). Correlation between microRNA-21 and expression of Th17 and Treg cells in microenvironment of rats with hepatocellular carcinoma. Asian Pac. J. Trop. Med..

[297] Dong L., Wang X., Tan J., Li H., Qian W., Chen J., Chen Q., Wang J., Xu W., Tao C. (2014). Decreased expression of microRNA-21 correlates with the imbalance of Th17 and Treg cells in patients with rheumatoid arthritis. J. Cell. Mol. Med..

[298] Go A.S., Chertow G.M., Fan D., McCulloch C.E., Hsu C.Y. (2004). Chronic kidney disease and the risks of death, cardiovascular events, and hospitalization. N. Engl. J. Med..

[299] Zoccali C., Goldsmith D., Agarwal R., Blankestijn P.J., Fliser D., Wiecek A., Suleymanlar G., Ortiz A., Massy Z., Covic A. (2011). The complexity of the cardio-renal link: Taxonomy, syndromes, and diseases. Kidney Int. Suppl..

[300] Johnson D.W., Dent H., Hawley C.M., McDonald S.P., Rosman J.B., Brown F.G., Bannister K., Wiggins K.J. (2009). Association of dialysis modality and cardiovascular mortality in incident dialysis patients. Clin. J. Am. Soc. Nephrol..

[301] Caillon A., Schiffrin E.L. (2016). Role of Inflammation and Immunity in Hypertension: Recent Epidemiological, Laboratory, and Clinical Evidence. Curr. Hypertens. Rep..

[302] Yao W., Sun Y., Wang X., Niu K. (2015). Elevated Serum Level of Interleukin 17 in a Population with Prehypertension. J. Clin. Hypertens..

[303] Cornelius D.C., Hogg J.P., Scott J., Wallace K., Herse F., Moseley J., Wallukat G., Dechend R., La Marca B. (2013). Administration of interleukin-17 soluble receptor c suppresses T H17 cells, oxidative stress, and hypertension in response to placental ischemia during pregnancy. Hypertension.

[304] Jafarzadeh A., Esmaeeli-Nadimi A., Nough H., Nemati M., Taghi Rezayati M. (2009). Serum levels of interleukin (IL)-13, IL-17 and IL-18 in patients with ischemic heart disease. Anadolu Kardiyol. Derg..

[305] Allam G., Abdel-Moneim A., Gaber A.M. (2018). The pleiotropic role of interleukin-17 in atherosclerosis. Biomed. Pharmacother..

[306] Baeten D., Sieper J., Braun J., Baraliakos X., Dougados M., Emery P., Deodhar A., Porter B., Martin R., Andersson M. (2015). Secukinumab, an Interleukin-17A Inhibitor, in Ankylosing Spondylitis. N. Engl. J. Med..

[307] Mease P.J., McInnes I.B., Kirkham B., Kavanaugh A., Rahman P., van der Heijde D., Landewé R., Nash P., Pricop L., Yuan J. (2015). Secukinumab Inhibition of Interleukin-17A in Patients with Psoriatic Arthritis. N. Engl. J. Med..

[308] Silfvast-Kaiser A., Paek S.Y., Menter A. (2019). Anti-IL17 therapies for psoriasis. Expert Opin. Biol. Ther..

